# Decitabine co-operates with the IL-33/ST2 axis modifying the tumor microenvironment and improving the response to PD-1 blockade in melanoma

**DOI:** 10.1186/s13046-025-03381-z

**Published:** 2025-05-02

**Authors:** Francesco Noto, Jacopo Mancini, Adriana Rosa Gambardella, Christina Curcio, Adele De Ninno, Sara Andreone, Carla Buccione, Maria Teresa D’Urso, Daniele Macchia, Anna Maria Pacca, Massimo Spada, Luca Businaro, Claudia Afferni, Fabrizio Mattei, Giovanna Schiavoni

**Affiliations:** 1 Department of Oncology and Molecular Medicine, Istituto Superiore Di Sanità, Rome, Italy; 2 Institute of Photonics and Nanotechnologies, Centro Nazionale Delle Ricerche (CNR-IFN), Rome, Italy; 3 Center of Animal Research and Welfare, Istituto Superiore Di Sanità, Rome, Italy; 4 National Center for Drug Research and Evaluation, Istituto Superiore Di Sanità, Rome, Italy

**Keywords:** IL-33, Decitabine, Melanoma, Immune checkpoint blockade, Tumor microenvironment, Tumor-immune crosstalk, DNA methylation, In vivo models, Organ-on-chip

## Abstract

**Background:**

IL-33 is an epithelial-derived alarmin with various roles in cancer. In melanoma, endogenous and exogenous IL-33 exert anti-tumor effects through the stimulation of several immune effector cells. In this study, we explored the combination of IL- 33 with Decitabine (DAC), a DNA methylation inhibitor that promotes immune recognition by re-activating silenced genes, for melanoma treatment.

**Methods:**

Multicellular spheroids, organ-on-chip technology and in vivo models were used to test the anti-tumor effects of IL-33 combined with DAC against mouse and human melanoma. Mice deficient for the IL-33 receptor ST2 (ST2^−/−^ mice) were employed to address the role of endogenous IL-33 signaling in DAC therapeutic response and tumor-immune crosstalk.

**Results:**

In multicellular spheroids of mouse and human melanoma cells, DAC alone inhibited tumor cell aggregation, suggesting its direct effect on tumor cells. In vivo, DAC combined with IL-33 reduced tumor growth and prolonged the survival of mice transplanted with melanoma cells, outperforming single treatments. Moreover, the combined DAC/IL-33 treatment was the most efficient in promoting immune recruitment (i.e., T cells and eosinophils) at the tumor site and induced the up-regulation of PD-1 resulting in better therapeutic response to PD-1 blockade in vivo. In a microfluidic-based competitive migration assay, DAC/IL- 33 treatment generated the strongest chemotactic response, attracting spleen cells from naïve wild-type, but not ST2^−/−^ mice, indicating that IL-33 signaling was crucial for immune cell recruitment. Accordingly, DAC failed to induce tumor immune infiltration and was ineffective in reducing tumor growth in ST2^−/−^ mice. In vivo, DAC increased the expression of ST2 and IL-33 at the tumor site, suggesting it may enhance endogenous IL-33 production. Methylation studies indicated that DAC increased the expression of IL-33 in mouse and human melanoma cells through demethylation of a transcription factor binding site located inside the *IL33* gene.

**Conclusions:**

Our findings indicate that DAC effectively co-operates with IL-33/ST2 axis against melanoma through immune cell recruitment and epigenetic regulation of gene expression, thus remodeling the tumor immune microenvironment to overcome resistance to PD- 1 inhibition.

**Supplementary Information:**

The online version contains supplementary material available at 10.1186/s13046-025-03381-z.

## Background

Interleukin- 33 (IL-33) is an epithelial-derived “alarmin” largely studied in the context of Th2-related immunopathologies and recently implied in cancer immunity [1]. Through the binding to its specific receptor ST2 expressed by most immune cells, IL-33 can stimulate a variety of immune reactions that can either support or contrast tumor growth [1]. In melanoma, a number of reports indicates that IL-33 promotes anti-tumor immune responses by recruiting and/or activating eosinophils, dendritic cells (DC), type- 2 innate lymphoid cells (ILC2), NK and CD8 T cells in vivo [2–7]. In the tumor microenvironment (TME), both tumor and stromal cells, such as fibroblasts, epithelial cells and some immune infiltrating cells are source of IL-33 [8]. Expression of IL-33 by tumor cells promotes CD8 T cell-mediated anti-tumor responses in various cancer models, including colorectal cancer [9], pancreatic ductal adenocarcinoma (PDAC) [10], melanoma and breast cancer [6] and is required for immune checkpoint blockade (ICB) efficacy [9, 11]. Moreover, tumor over-expression or exogenous administration of IL-33 can improve the therapeutic response to ICB in tumor-bearing mice [11–14]. Due to its ability to tailor the tumor immune microenvironment, IL-33 represents a promising candidate for combinatorial anti-cancer therapies.

Epigenetic modifications play important roles in melanoma progression, promoting immune escape and metastatic spread. In melanoma, aberrant DNA hypermethylation is frequently observed, resulting in the silencing of several genes involved in cell cycle regulation, apoptosis, tumor growth and drug resistance [15]. Epigenetic drugs, such as DNA hypomethylating agents (DHAs) have been employed in pre-clinical studies as a strategy to manipulate the transcriptional program of melanoma cells [16]. DHAs can potentiate immune recognition through the up-regulation of class I and co-stimulatory molecules, the promotion of CD8 T-cell responses, and the regulation of immune checkpoint molecules [15, 17, 18]. These evidences have provided rationale for using DHAs to improve the efficacy of immunotherapy in pre-clinical models of melanoma [19–21]. The DHA 5-Aza-2’-Deoxycitidine (Decitabine, DAC; DrugBank ID: DB01262) is an inhibitor of the DNA methyl transferase enzymes (DNMTi), whose action results in the restoration of previously silenced genes, such as oncosuppressors. In melanoma models, DAC cooperates with IL- 12 [22], type I IFNs [20] or ICAM-1 antibody drug conjugates [23] to promote anti-tumor efficacy by sensitizing to direct tumor killing and by remodeling the TME. Moreover, combination of DAC and type I IFNs enhance the therapeutic benefit of DNA vaccines in melanoma-bearing mice [24, 25], suggesting that combinatorial regimen of DAC with immunostimulating cytokines are a promising strategy for ameliorating anti-tumor response.

In this study, we explored the potential synergistic role of DAC with IL-33/ST2 axis against melanoma. We show that the combination of DAC with IL-33 remodels the TME and ameliorates immunotherapy response to PD-1 blockade in melanoma-bearing mice. We also show that DAC epigenetically induces IL-33 expression and that IL-33/ST2 tumor-immune axis is crucial for immune migratory response and anti-tumor effects of DAC.

## Methods

### Cell lines and chemicals

Murine B16.F10 melanoma cells were purchased from the American Type Cell Collection (ATCC, Manassas, VA, US; CRL- 6475). Human A375M melanoma cells (CVCL_B222) were kindly provided by Dr. Alessandra Carè (Istituto Superiore di Sanità, Rome, Italy). Mouse and human melanoma cells were cultured in Dulbecco’s Modified Eagle’s Medium (DMEM) supplemented with 10% of Fetal Bovine Serum (FBS) 1% glutamine, 1% antibiotic–antimycotic (all from Euroclone, Pero, MI, Italy). Cell lines were routinely tested for morphology, absence of mycoplasma and passaged for no more than 4 times from thawing. Decitabine (A3656 5-Aza- 2’-deoxycytidine; DAC) cisplatin (C2210000; CDDP) and doxorubicin hydrochloride (D1515; DOXO) were purchased from Sigma Aldrich (Saint Louis, MO, US). Recombinant mouse IL-33 (rmIL-33) carrier-free (amino acids Ser109-Ile266) was purchased from Biolegend (San Diego, CA, US). Recombinant human IL- 33 (rhIL-33) was purchased from Cell Guidance Systems (Cambridge, UK).

### Tumor spheroid assay

Multicellular tumor spheroids from murine B16.F10 and human A375M melanoma cells were generated in ultralow attachment surface 96 well plates (Corning, NY, US). Five hundred cells were cultured in 100 μL of complete DMEM. B16.F10 cells were treated with 0.1 μM DAC, 100 ng/mL rmIL-33, alone or in combination. A375M cells were treated with 0.25 μM DAC 100 ng/mL rhIL-33, alone or in combination. Five wells per experimental conditions were set. One representative visible channel microphotograph has been captured per each well at different time of culture by using an EVOS-FL microscope (Life Technologies, Carlsbad, CA, US). The acquired replicate images were then subjected to particle analysis quantification by using the homonym ImageJ plugin (https://imagej.net/ij/). The area (expressed in μm^2^, used as size) of all spheroid displayed in each microphotograph was then automatically achieved by this plugin. The histograms represent the mean area values taken from all the spheroid units present in the five wells (microphotographs) per each experimental condition. An optimal thresholding algorithm was chosen for each region, depending on visible light dispersion and distribution. Spheroid area was used as a representative morphometric parameter.

### Mice and in vivo treatments

C57BL/6 J mice (Envigo, San Pietro al Natisone, UD, Italy) and ST2^−/−^ mice on a C57BL/6 J background (kindly provided by Dr. Andrew N. McKenzie) were housed in the animal facility at the Istituto Superiore di Sanità (Rome, Italy). For the evaluation of the combined DAC/IL-33 effect on melanoma growth, C57Bl/6 mice were injected subcutaneously with 0.8 × 10^6^ B16.F10 cells. When the tumor reached 3 mm of diameter (approximately 6–7 days post-transplant), mice were injected once intraperitoneal (i.p.) with DAC (1 mg/kg,) and/or intratumoral (i.t.) with rmIL-33 (0.4 μg per mouse). IL-33 injections were repeated for 4 consecutive days for a total of five administrations. For the evaluation of the efficacy of PD-1 blockade, melanoma-bearing mice treated with DAC plus IL-33 received three i.p. injections of 250 μg/mouse PD-1 mAb (clone 29 F.1 A12, Leinco Technologies, Saint Louis, MO, US), every three days starting from the last day of IL-33 administration. Control groups included mice receiving IL-33 alone, DAC alone, PD-1 mAb alone, IL-33 plus PD-1 mAb, or DAC plus PD-1 mAb. For the evaluation of chemo-drug response, C57BL/6 J wild-type and ST2^−/−^ melanoma-bearing mice received a single injection of DAC (1 mg/kg, i.p.), CDDP (2.5 mg/kg i.t.) or DOXO (2 mM i.t.). Tumor growth in all mice was measured twice a week using a caliper. Where indicated, mice were sacrificed at appropriate times for ex vivo analyses.

### Flow cytometry analysis of tumor-infiltrating immune cells

For flow cytometry analysis, melanoma explants were cut into small fragments using curved scissors and then digested in a medium containing type III collagenase (1 mg/ml; Worthington Biochemicals, Lakewood, NJ, US) and DNase I (325 KU/mL; Sigma) for 30 min at room temperature in agitation, followed by EDTA (0.1 M pH 7.2) for additional 5 min. The homogenate was then passed through a cell strainer and the resulting suspension was treated with Ammonium-Chloride-Potassium (ACK) lysis buffer (8.26 g NH_4_Cl, 1 g KHCO_3_, 0.037 g EDTA 0,1 M in 1 L of distillated sterile H_2_O) to eliminate erythrocytes. Cells were incubated with LIVE/DEAD® Fixable Near-IR Dead Cells Stain Kit (Life technologies) and then labelled with fluorescent antibodies (mAbs). We used these following mAbs: anti-CD45 (clone 30-F11) and anti-Siglec-F (clone E50 - 2440) from BD Bioscience (Franklin Lakes, NJ, US); anti-CD3 (clone 145 - 2 C11), anti-CD4 (clone GK1.5), anti-CD8 (clone 53–6.7), anti-CD11b (clone M1/70), anti Ly6G (clone 1 A8), all from Biolegend; anti-CD279 (PD-1; clone J43) from eBioscience (San Diego, CA, US). Biotinylated antibodies were detected by Streptavidin PercP-Cy5.5 or eFluor450 (eBioscience). Samples were run on a Gallios flow cytometer and analyzed with the Kaluza Analysis Software (Beckman Coulter). Cell populations were defined as follows: CD45^+^CD3^+^ CD11b^−^CD4^+^CD8^−^ (CD4 T cells); CD45^+^CD3^+^CD11b^−^CD4^−^CD8^+^ (CD8 T cells); CD45^+^CD11b^hi^SiglecF^hi^Ly6G^−^ (eosinophils); CD45^+^CD11b^hi^SiglecF^−^Ly6G^+^ (granulocytic myeloid-derived suppressor cells, G-MDSC).

### Quantitative reverse transcription-PCR (qPCR)

Total RNA was extracted from tumor tissue by using Trisure reagent (cat n° BIO- 38033, Meridian Bioscience, Memphis, TN, US). Messenger RNA was reverse transcribed by means of Tetro cDNA Synthesis kit (BIO.65043, Meridian Bioscience). Quantitative reverse transcription-PCR (qPCR) was performed using SensiFAST SYBR Lo-ROX Kit containing the fluorescent dye SYBR Green (BIO- 94020, Meridian Bioscience). Forward and reverse primers (Table S1) were purchased from Eurofins Genomics (Louisville, KY, US). The conditions of the real-time PCR reaction were: 15 s at 95 °C, 30 s at 60 °C and 45 s at 72 °C for 45 cycles using the Applied Biosystem™ 7500 Fast Real-Time PCR System apparatus. Specificity and quality of amplicons in each sample were detected by dissociation curves elaborated by the applicative software of the machine (7500 Software, version 2.0.6). Triplicates were performed for each experimental point. For quantitation, threshold cycle (CT) values were determined by the Sequence Detection System software (Applied Biosystems, Waltham, MA, US), and data were presented as fold expression vs. HPRT housekeeping gene (2-ΔCt method).

### Isolation of mouse spleen cells and human peripheral blood mononuclear cells

Briefly, spleens were removed from naïve C57Bl/6 J wild-type or ST2^−/−^ mice and sieved through a cell strainer with the plunger of a syringe for mechanical dissociation. The resulting cell suspension was treated with ACK lysis buffer to eliminate red blood cells. Peripheral blood mononuclear cells (PBMCs) were obtained from healthy donor buffy coats by Ficoll-Hypaque density-gradient centrifugation by using Lymphosep, Lymphocyte Separation Media (L0560, Biowest, Nuaillé, France).

### Competitive migration assay in 3D microfluidic chips

Microfluidic chips for co-culture competitive assay were fabricated in polydimethylsiloxane (PDMS), as previously described [20, 26]. The devices consist of two center end closed channels adjacent to two cell culture compartments. The two main culture channels and the center channel were connected by micro-sized channels, as depicted in Supplementary Fig. 1. Melanoma cells (B16.F10 cells or A375M cells) were labeled with the membrane dye PKH67 Green Fluorescence Cell Linker (MINI67, Sigma Aldrich) and then resuspended in ice cold Matrigel (2 mg/ml; Corning). Where indicated, DAC and IL-33 were added single or combined to the melanoma cell-Matrigel mixture. For mouse cells, DAC was used at 0.1 μM and rmIL- 33 at 100 ng/ml. For human cells, DAC was used at 0.25 μM and rhIL-33 at 100 ng/ml. The melanoma cell-Matrigel mixtures (2 × 10^4^ cells in 5 μl) were loaded in the two narrow lateral chambers (see Supplementary Fig. 1) and the devices were placed at 37 °C for 30 min to allow gel solidification. In a second step, immune cells (mouse spleen cells or human PBMCs) were labelled with PKH26 Red Fluorescence Cell Linker (MINI26, Sigma Aldrich), resuspended in RPMI complete medium and loaded (1 × 10^6^ cells in 100 μl) in the central chamber of the device. As a final step, the reservoirs of the lateral chambers were filled with medium and the devices were placed in a 37 °C, 5%CO_2_ incubator for 48 h. Phase contrast, visible and fluorescence photomicrographs were generated by using EVOS-FL fluorescence microscope (Life Technologies) provided with built-in imaging software for imaging overlays. Quantitative fluorescence analysis was carried out with ImageJ software (https://imagej.net/ij/).

### Detection of CD8 T cells in 3D microfluidic chips by fluorescence analysis

PKH26 Red-labelled PBMCs were loaded in the chip together with Matrigel-embedded unlabeled A375M cells exposed to the various treatments in a competitive setting, as described above. After 48 h co-culture, the medium was aspirated from the lateral and central fluidic chambers, and channels were washed with PBS followed by 1% bovine serum albumin (BSA) in PBS to remove residual PBMCs not infiltrated in the Matrigel chambers containing melanoma cells. After the washes, staining was carried out with anti-human CD8 Alexa Fluor 488 (clone OKT8; eBioscience) and anti-human CD3 biotin (clone OKT3; Biolegend) followed by Streptavidin Alexa Fluor 647 (eBioscience) for 45 min at 4 °C. The devices were fixed with a 2% paraformaldehyde (PFA) and 1% glutaraldehyde solution for 20 min and finally stained with DAPI (4',6-Diamidino- 2-Phenylindole, Dihydrochloride; Invitrogen, Waltham, MA, US) for 45 min at 4 °C for nuclei labelling. Detection of CD3^+^CD8^+^ T cells and their spatial interaction with A375M cells in the Matrigel chambers were performed by laser scanning confocal microscopy (LSCM). The Z-Stack/XY planes 3D microphotographs were acquired on a LSCM station Zeiss LSM 900 (Carl Zeiss GmbH, Jena, Germany) in Airyscan mode. Excitation light was obtained by using diode lasers 405 nm, 488 nm, 647 nm and 555 nm. Images and Z-stack/XY planes were obtained and processed by the Zen Blue (v3.2) software (Carl Zeiss). ImageJ was employed for Z-stack/XY planes processing.

### Methylation Specific qPCR (MSP-qPCR) in melanoma cells on IL33 gene promoters

B16.F10 and A375M melanoma cells were seeded in 6 well plates (Corning) at a concentration of 5 × 10^5^ in 2 ml of culturing medium. Mouse and human melanoma cells were treated with DAC 0.1 μM and 0.25 μM, respectively, or left untreated. After 24 h of culture, cells were harvested and genomic DNA was extracted from melanoma cells by Quick DNA™ MiniPrep (CAT N° D3024, Zymo Research, Irvine, CA, US). Sodium bisulfite treatment of genomic DNA to convert unmethylated cytosine into uracil was carried out using EZ DNA Methylation™ Kit (Cat n° D5001, Zymo Research) according to manufacturer’s instructions. MSP-qPCR primers were designed using the web tool MethPrimer (https://www.urogene.org/cgi-bin/methprimer/methprimer.cgi), sequences listed in Table S2). These primers were selected based on the presence of CpG islands, detected by The CpG Island search tool into the JavaScript toolbox Sequence Manipulation Suite (https://www.compgen.uni-muenster.de/tools/sms2/cpg_islands.html). Real time qPCR was performed on bisulfite converted cDNA with an Applied Biosystems™ 7500 Fast Real-Time PCR System (Life Technologies) by using the methylated or unmethylated primer pairs, which distinguish between methylated or unmethylated sequence of the P1 (mouse) or P (human) promoters. The *Il33* gene sequence shows that there are three different promoter regions (P1, Promoter 2, and Promoter 3) positioned in different regions into this gene (Supplementary Fig. 2). We analyzed methylation status of a sequence into the P1 promoter (but not Promoter 2 and Promoter 3) sequence because of its optimal position at the 5’ region of 3 *Il33* isoforms (ENSMUST00000120388, ENSMUST00000144528, ENSMUST00000025724), spanning all the exons. Therefore, P1 sequence represents a potential suitable promoter for *Il33* gene because of its upstream (5’ region) location referring to the entire gene sequence, and also covers the exon 1 (Supplementary Fig. 2). Similarly, the unique P promoter for two *IL33* gene isoforms (ENST00000417746, ENST00000682010) is located upstream to the gene sequence and spans the *IL33* exon 1 region (Supplementary Fig. 2). The conditions of real time MSP-qPCR reaction were as follows: 15 s at 95 °C, 30 s at 60 °C and 45 s at 72° for 40 cycles. The calculation of the methylation/demethylation status of human and murine *IL33* gene promoters was carried out as previously described [17]. Briefly, the percentage of methylation (% Meth) was defined as ratio between methylated fractions and the sum of methylated plus unmethylated fractions and data were reported as %. Vice versa, the percentage of demethylation (% Demeth) was defined as ratio between unmethylated fraction and the sum of methylated plus unmethylated fraction reported as %. The aforementioned normalized % Meth and % Demeth percent values (Fig. 5) refer to the analyzed *Il33* P1 and *IL33* P gene promoters (Supplementary Fig. 2).

### MTS assay

One thousand B16.F10 and A375M melanoma cells were seeded in 200 µL of fresh medium in flat 96-well plates (Corning) in the absence or presence of graded concentrations of DAC, IL-33 (100 ng/mL) or their combination. After 24 h, 48 h and 72 h of culture, 20 µL of 3-(4,5-dimethylthiazol- 2-yl)− 5- (3-carboxymethoxyphenyl)− 2-(4-sulfophenyl)− 2H-tetrazolium (MTS; CellTiter 96™ AQ_ueous_ Nonradioactive Cell Proliferation Assay Kit, Promega, Madison, WI, US) were added to each well and cells further incubated for 1 h at 37 °C in 5% CO_2_. Absorbance was measured in a spectrophotometer at a wavelength of 490 nm.

### Statistical analysis

Mann–Whitney test was used for the nonparametric analysis of differences between two groups. One-way ANOVA analysis of variance followed by post hoc testing (Tukey) was used to compare means among multiples groups. Two-way ANOVA analysis of variance was used to compare tumor growth curves. Log-rank Mantel-Cox test was used for the analysis of survival curves. Values were considered significant when the probability was below the 5% confidence level (*p* ≤ 0.05).

## Results

### Effects of combined DAC/IL-33 treatment on melanoma growth and immune landscape

**Fig. 1:**
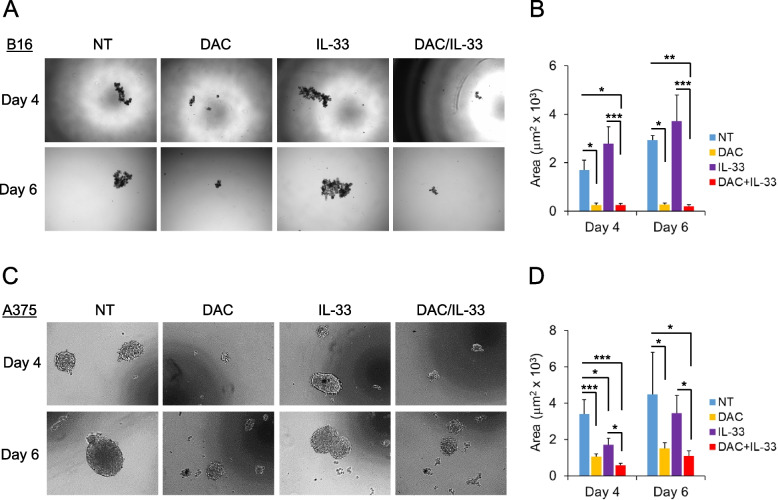
Effect of DAC in combination with IL-33 on the growth of melanoma spheroids. **A** Murine B16.F10 melanoma cells were seeded on a 96-well ultralow attachment plates without treatment (NT) or in the presence of DAC (0.1 mM), rmIL-33 (100 ng/mL) or with the combination of DAC/IL-33. Microphotographs show the architecture of 3D spheroids after 4 and 6 days of culture time. **C** Human A375M melanoma cells were seeded on a 96-well ultralow attachment plates without treatment (NT) or in the presence of DAC (0.25 mM), rhIL-33 (100 ng/mL) or with the combination of DAC/IL-33. Microphotographs show the architecture of 3D spheroids after 4 and 6 days of culture time. On the right side, graphical representation of the analysis of the mean area (expressed as mm^2^ x 10^3^, where the latter number is a scale factor) of each condition (5 replicates per condition) ± SD in (**B**) B16.F10 and (**D**) A375M spheroids.*P< 0.05, ** *P*< 0.01, *** *P*< 0.001

We aimed to assess whether the combination with DAC could increase the anti-tumor efficacy of IL-33. We first evaluated the direct anti-tumor effects of the combination of DAC with IL-33 on mouse and human melanoma 3D cultures. To this end, murine B16.F10 and human A375M melanoma cells were seeded in ultra-low attachment plates in medium alone or in the presence of DAC, IL-33 or the combo DAC/IL-33. In untreated controls, formation of defined B16.F10 (Fig. 1A) and A375M (Fig. 1C) melanoma spheroids in each well was visible after four days of incubation. Exposure of melanoma cells to IL-33 did not interfere with the formation of either mouse or human melanoma spheroids (Fig. 1B, D). In contrast, in the presence of DAC, either alone or combined with IL-33, spheroid formation was inhibited in both B16.F10 (Fig. 1A, B) and A375M (Fig. 1C, D) melanoma cells up to day 6. MTS assays on 2D cultures confirmed that DAC exhibited dose-dependent anti-proliferative effects on both mouse B16.F10 (Supplementary Fig. 3 A) and human A375M (Supplementary Fig. 3B) melanoma cells independently of IL-33. These data indicate that DAC, but not IL-33, has direct anti-proliferative effects against melanoma cells.

**Fig. 2:**
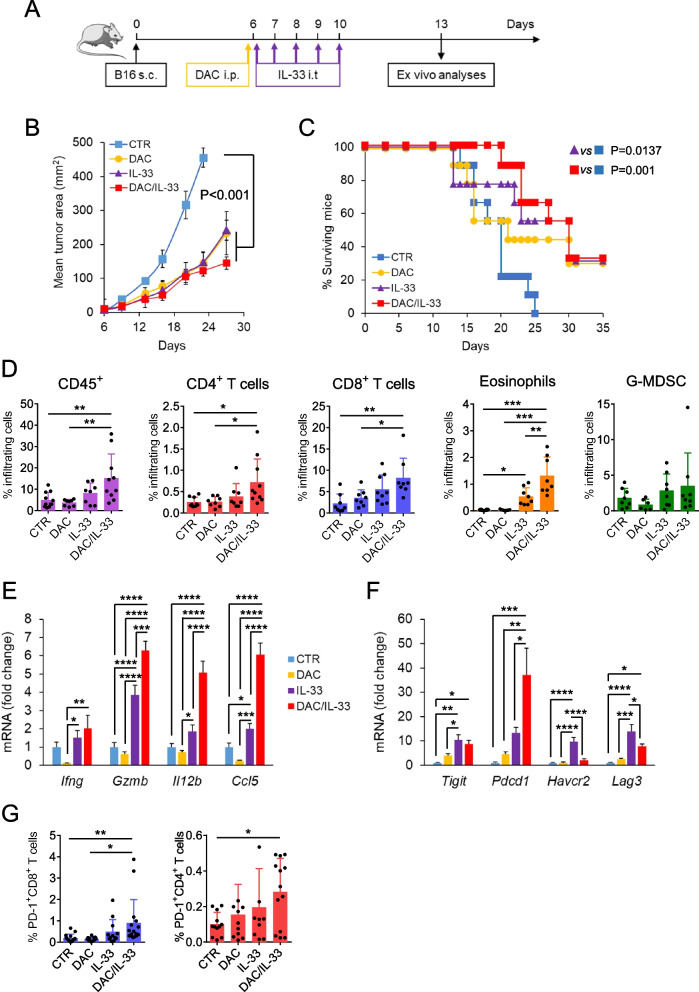
Combined DAC/IL-33 in vivo treatment on melanoma growth and tumor microenvironment. **A** Schematic representation of experimental protocol. C57BL/6 mice were injected subcutaneously with B16.F10 melanoma cells (0.8 x 10^5^) and then treated with DAC (1 mg/kg i.p.), rmIL-33 (0.4 mg/mouse i.t.) singly or combined. Control mice (CTR) received PBS. *Ex vivo* analyses were carried out on day 13. **B** Tumor growth in the indicated groups. Mean tumor area of individual mice (*n*= 9) ± SD is shown. **C** Kaplan Meier Plot representing percentage of surviving mice within each group. **D** Flow cytometry analysis of indicated tumor infiltrating immune cells at day 13. Data shown the mean value of individual mice (*n*=7–12) ± SD. **E** Chemokine/cytokine and (**F**) immune checkpoints expression in melanoma tumors by real time PCR. Data are expressed as mean fold change of mRNA expression vs control of individual mice (*n*= 7) ± SD. **G** Percentage of PD-1 expressing tumor-infiltrating CD8-T and CD4-T cells. Data represent the mean value of individual mice (*n*=12–16) ± SD. *P< 0.05, ** *P*< 0.01, *** *P*< 0.001, ****P< 0.0001

Next, we evaluated whether the combination with DAC could increase the anti-tumor efficacy of IL-33 in vivo. In melanoma-bearing mice, a single injection of DAC (i.p.) in combination with five intratumoral (i.t.) injections of IL-33 (Fig. 2A) reduced tumor growth significantly, with respect to control mice, although not with respect to mice exposed to single treatment (Fig. 2B). However, mice exposed to DAC/IL-33 treatment exhibited the most significant increase in survival rate with respect to controls (Fig. 2C). We analyzed the immune infiltrate in melanoma-bearing mice three days after the last cytokine administration and found that DAC/IL-33 treatment significantly promoted the recruitment of immune (CD45^+^) cells in the tumor (Fig. 2D). Combined DAC/IL-33, but not the single treatments, induced significant increase in tumor-infiltrating CD4 and CD8 T cells with respect to untreated mice (Fig. 2D). Moreover, the accumulation of eosinophils in melanoma from DAC/IL-33 treated mice was significantly superior with respect to single treatments and untreated controls (Fig. 2D). In contrast, the frequencies of G-MDSC were not changed (Fig. 2D). Gene expression analysis revealed that DAC and IL-33 synergized to up-regulate Th1-related effector molecules, including Granzyme B (*Gzmb*), IL- 12 (*Il12b*) and the T cell-attracting chemokine CCL5 (Fig. 2E). IFN-γ expression also increased in tumors of mice exposed to DAC/IL-33, albeit not in a synergistic manner (Fig. 2E). We sought to investigate whether this combined treatment could increase the expression of immune checkpoints within the tumor microenvironment. We found that DAC and IL-33 strongly synergized to up-regulate PD-1, but not other molecules analyzed (i.e., TIGIT, Tim-3 or LAG-3; Fig. 2F). Flow cytometry analysis confirmed significantly higher percentages of PD-1-expressing CD4 and CD8 T cells in melanoma tumors from mice exposed to the combo DAC/IL-33 treatment, but not from those receiving single treatments, with respect to untreated animals (Fig. 2G). DAC/IL-33 treatment was also superior to DAC alone in increasing the percentage of PD-1-expressing CD8 T cells (Fig. 2G). In contrast, we did not observe significant increase in surface TIGIT, Tim-3 or LAG-3 in either CD4 or CD8 T cells by any treatment (Supplementary Fig. 4).

### DAC/IL-33 unleashes the response to PD-1 blockade in melanoma-bearing mice

**Fig. 3:**
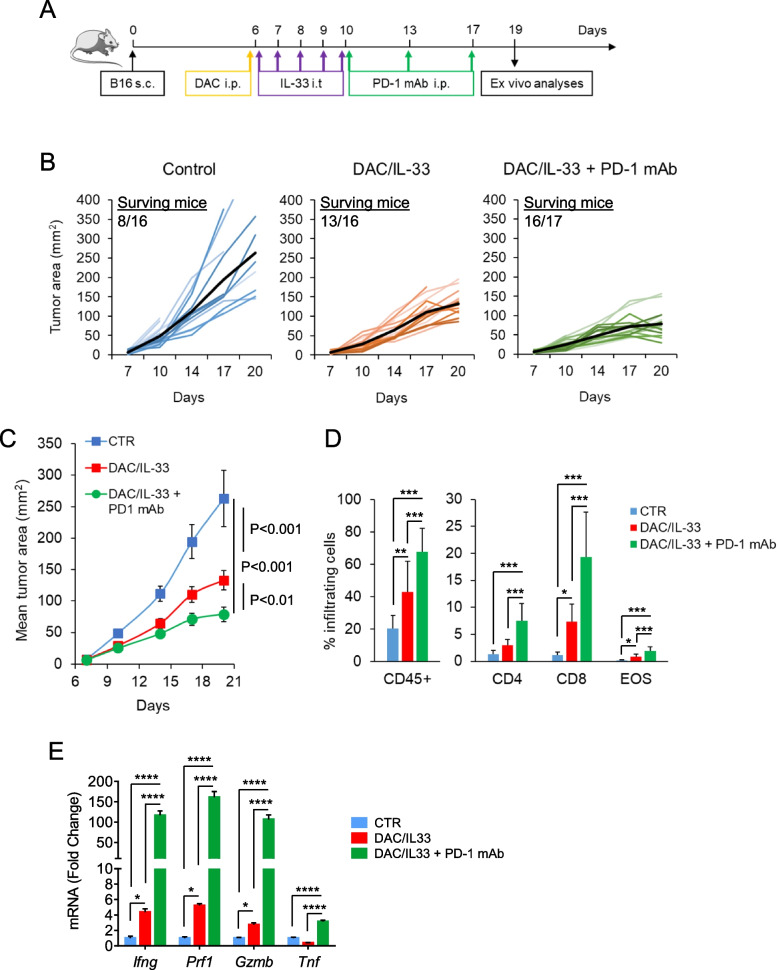
DAC/IL-33 treatment ameliorates the therapeutic response to PD-1 blockade in melanoma bearing mice. **A** Schematic representation of experimental protocol. C57BL/6 implanted with B16.F10 melanoma cells (0.8 x 10^5^) were treated with DAC plus IL- 33 (as in Fig. 2) with or without PD-1 mAb (250 m i.p.). Ex vivo analyses were carried out on day 19. **B**, **C** Tumor growth. **B** Tumor area of individual mice. Black bold line represents the mean for every group. The number of surviving mice at day 19 is depicted. **C** Mean tumor area of individual mice (*n*= 16) ± SD. **D** Flow cytometry analysis of tumor infiltrating cells. Data show the mean value of individual mice (*n*=9–15) ± SD. **E** Expression of indicated cytokines in melanoma tumors by real time PCR. Data are expressed as mean fold change of mRNA expression vs control of individual mice (*n*= 7) ± SD. *P< 0.05, ** *P*< 0.01, *** *P*< 0.001 ****P< 0.0001

The above results showing induction of PD-1 expression in the TME by DAC/IL-33 prompted us to assess whether this combined treatment could increase the therapeutic response to PD-1 blockade in mice. To this aim, mice exposed to DAC/IL-33 were injected i.p. with PD-1 mAb (clone 29 F.1 A12) every three days starting from the last day of IL-33 administration (Fig. 3A). As expected, PD-1 mAb treatment alone failed to reduce melanoma growth (Supplementary Fig. 5 A), confirming that B16.F- 10 melanoma is unresponsive to blockade of this immune checkpoint [27]. Moreover, PD-1 mAb combined with either IL-33 or DAC alone failed to synergistically reduce tumor growth (Supplementary Fig. 5 A, B). By contrast, pre-exposure to DAC/IL-33 unleashed the anti-tumor response to PD-1 mAb in melanoma-bearing mice, as shown by significant tumor growth delay and ameliorated survival with respect to DAC/IL-33 and control (Fig. 3B, C). The beneficial effect of PD-1 blockade in DAC/IL-33 treated mice correlated with a significant increase in immune (CD45^+^) tumor-infiltrating cells, particularly CD4 and CD8 T cells and eosinophils (Fig. 3D) and with increased expression of IFN-γ, granzyme B (*Gzmb*), perforin (*Prf1*) and tumor necrosis factor-α (*Tnf*, Fig. 3E), indicating activation of effector anti-tumor immunity. These data indicate that DAC/IL-33 is effective in unleashing the anti-tumor response to PD-1 blockade.

### DAC epigenetically activates *IL33* expression and requires IL-33/ST2 axis for anti-tumor efficacy

**Fig. 4:**
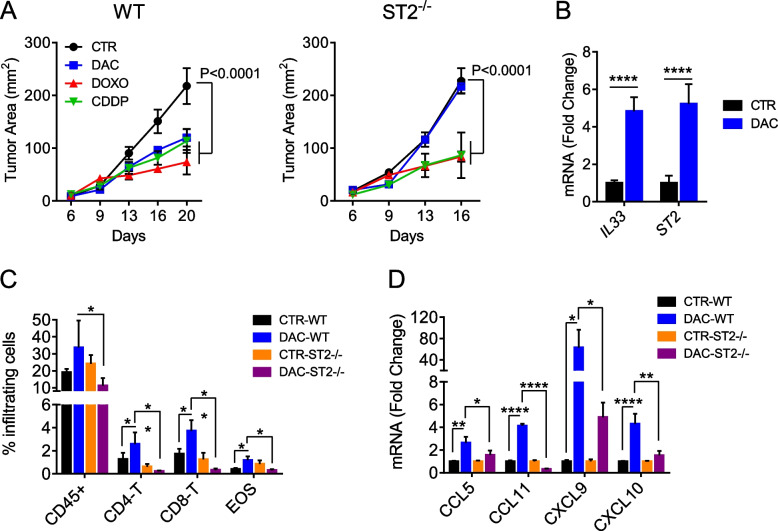
Role of IL-33/ST2 axis in the antitumor efficacy of DAC. **A** C57BL/6 wild-type (WT) and ST2^-/-^ mice implanted with B16.F10 melanoma cells (0.8 x 10^5^) were treated with DAC (1 mg/kg i.p.), CCDP (2.5 mg/kg i.t.) or DOXO (2 mM i.t.). Mean tumor area of individual mice (*n*=5–10) ± SD is shown. **B** Expression of *IL33* and *ST2* in melanoma tumors of C57BL/6 WT mice 3 days after DAC treatment. Data are expressed as mean fold change of mRNA expression *vs* control of individual mice (*n*= 5) ± SD. ****P< 0.0001. **C** Immune infiltrates in C57BL/6 WT and ST2^-/-^ mice 3 days after DAC treatment. Data show the mean value of individual mice (*n*= 5) ± SD. **D** Expression of indicated chemokines in melanoma tumors C57BL/6 WT and ST2^-/-^ mice 3 days after DAC. Data are expressed as fold change of mRNA expression vs control of individual mice (*n*= 5) ± SD. *P< 0.05, ** *P*< 0.01, *** *P*< 0.001 ****P< 0.0001

To explore the possible mechanisms underlying the cooperative action between DAC and IL-33, we analyzed the requirement for IL-33 signaling for DAC anti-tumor efficacy in vivo in mice lacking the IL-33 specific receptor ST2. ST2^−/−^ and wild-type (WT) melanoma-bearing mice were treated with DAC or, as a control, other anti-neoplastic drugs, namely cisplatin (CDDP) and the immunogenic drug doxorubicin (DOXO). While CDDP and DOXO were effective in delaying melanoma growth in both WT and ST2-deficient mice, the anti-tumor efficacy of DAC was completely lost in ST2^−/−^ mice (Fig. 4A). These data indicate that DAC requires endogenous IL-33 signaling for its antitumor efficacy. To explore whether DAC positively modulates the IL-33/ST2 axis in the tumor microenvironment, we assessed *IL33* and *ST2* expression in the TME of mice early after exposure to this epigenetic modifier drug (i.e., 3 days). We found significantly increased expression of both *IL33* and its receptor *ST2* in melanoma tissues from WT mice exposed to DAC treatment with respect to untreated controls (Fig. 4B). Moreover, at this time point we observed increased frequencies of tumor-immune infiltrates, particularly CD4-T and CD8-T cells and eosinophils in DAC treated WT but not ST2^−/−^ mice (Fig. 4C). These traits correlated with up-regulated levels of chemokines attracting T cells (CCL5, CXCL9, CXCL10) and eosinophils (CCL11) in DAC treated WT with respect to ST2^−/−^ mice (Fig. 4D). These observations suggest a correlation between immune recruitment and induction of *IL33* and *ST2* expression by DAC in the TME.

**Fig. 5:**
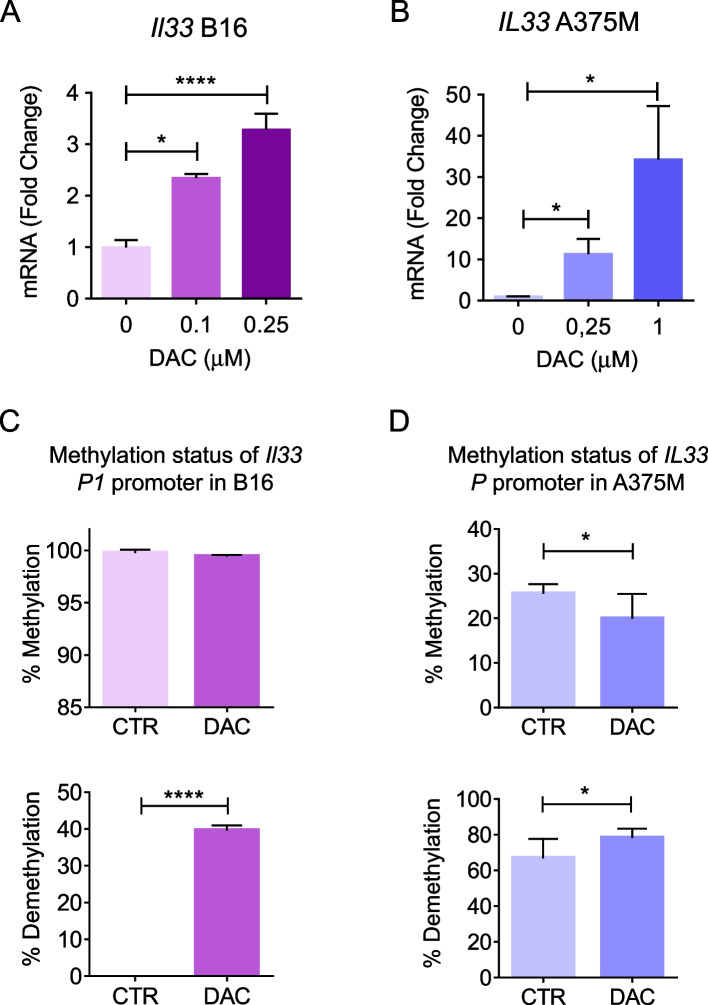
DAC epigenetically regulates *IL33* gene expression in melanoma cells. Expression of *IL33* in (**A**) murine B16.F10 and (**B**) human A375M melanoma cells after exposure to increasing doses of DAC by real time PCR (24 h). Histograms represent mean fold change of mRNA expression vs control in culture replicates ± SD. *P< 0.05, **** *P*< 0.0001. MSP-qPCR analysis on (**C**) murine *il33* and (**D**) human *IL33* gene promoters. Histograms indicate the percentages of methylation and demethylation status of P1 promoter region in B16.F10 (**C**) and P promoter region in A375M melanoma cells (**D**) 24 h after exposure to, respectively, 0.1 mM and 0.25 mM DAC as opposed to untreated cells (CTR). *P< 0.05, ****P< 0.0001

Since tumor cells can be a source of IL-33 [8, 9], we analyzed whether DAC could directly induce IL-33 expression in melanoma cells. Exposure to DAC dose-dependently increased the expression of *IL33 gene *in mouse B16.F10 (Fig. 5A) and human A375M (Fig. 5B) melanoma cells with respect to controls. Given its DNA methylation inhibition activity, we conducted methylation-specific quantitative PCR (MSP-qPCR) analysis of *il33* gene promoter P1 in B16.F10 and *IL33* gene promoter P (Supplementary Fig. 2) in A375M melanoma cells treated with DAC. After 24 h exposure to 0.1 μM DAC, B16.F10 melanoma cells exhibited significant demethylation (7-fold increase with respect to untreated cells) of *il33* gene promoter P1 (Fig. 5C). Similarly, in A375M we found decreased methylation and increased demethylation in the *IL33* gene promoter P1 following treatment with 0.25 μM DAC (Fig. 5D). These data indicate that DAC epigenetically activates *IL33 *gene expression in tumor cells and that functional endogenous IL-33/ST2 axis is required for its anti-tumor efficacy in vivo.

### DAC and IL-33/ST2 axis cooperate to promote immune cell recruitment to the tumor site

**Fig. 6:**
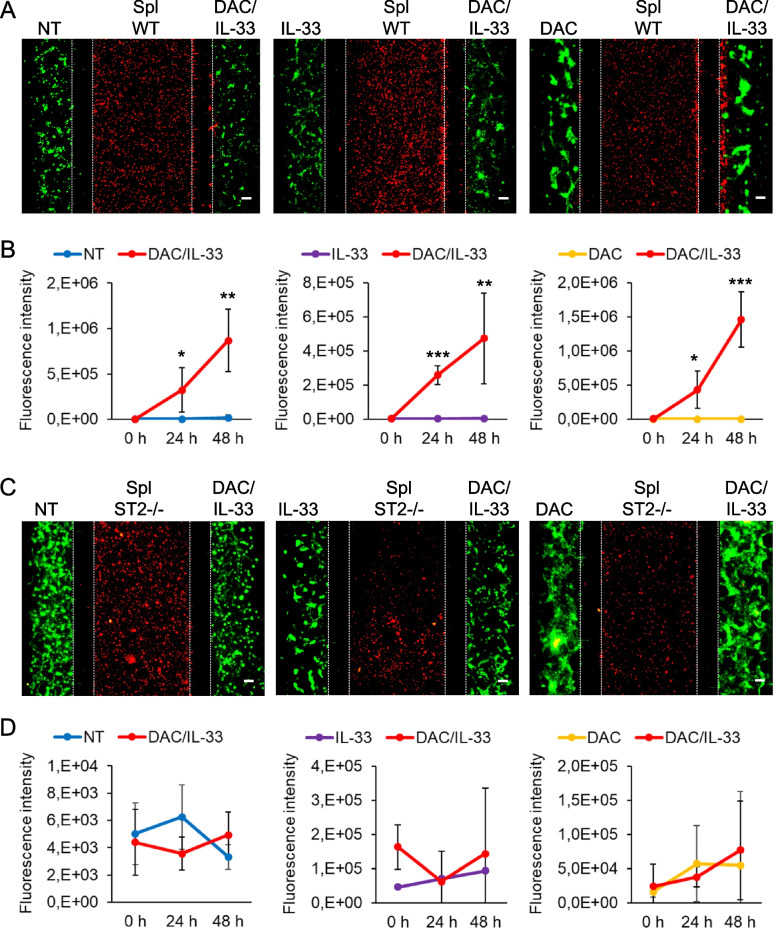
DAC and IL-33/ST2 axis cooperate to promote tumor-immune crosstalk in a 3D microfluidic system. Microfluidic devices were used to analyze the competitive migration of spleen cells from (**A**) C57BL/6 WT or (**C**) ST2^-/-^ mice towards melanoma cells exposed to DAC/IL-33. PKH26-labeled (red) spleen cells were loaded in the central chamber of microfluidic devices. PKH67-labeled (green) B16.F10 melanoma cells embedded in Matrigel containing the treatments were placed in lateral chambers. In every device the condition DAC/IL-33 was confronted with untreated condition (NT, left), IL-33 alone (central) or DAC alone (right). Fluorescence images were obtained after 48 h of culture. Red fluorescence represents displacement of (**A**) WT and (**C**) ST2^-/-^ spleen cells towards melanoma chambers (green) containing the indicated treatments. Discontinued vertical white lines depict microchannel area. Scale bars = 100 mm. Quantitative analysis of red fluorescence intensity in the tumor chambers reflecting the preferential migration of (**B**) WT and (**D**) ST2^-/-^ spleen cells towards DAC/IL-33 treated vs untreated (left), IL-33 treated (middle) or DAC treated (right) melanoma cells. Data show the mean ± SD of several fields of at least 3 devices per experimental condition. *P< 0.05, ** *P*< 0.01, *** *P*< 0.001

We further explored the cooperative action of DAC with the IL-33/ST2 axis in the tumor-immune crosstalk by means of 3D microfluidic chip-based competitive assays [3, 20, 26, 28, 29]. In this setting, PKH67 green-labeled B16.F10 melanoma cells embedded in Matrigel are loaded in the two opposite chambers alone or with added IL-33, DAC or the combo DAC/IL-33, and confronted for their capacity to attract PKH26 red-labeled spleen cells from naïve mice, either ST2^−/−^ or WT, loaded into the middle fluidic (Supplementary Fig. 1). Migration and infiltration of spleen cells towards either the left or right chamber through the connecting microchannels was evaluated by fluorescence microscopy after 48 h. Images (Fig. 6A) and quantitative analysis of red fluorescence in the melanoma chambers (Fig. 6B) show that DAC/IL-33 was the most effective treatment inducing the infiltration of WT spleen cells, revealed by preferential displacement of the immune cells towards the right chamber containing melanoma cells exposed to the combo treatment as opposed to the left chamber containing either single drug-treated (IL-33 or DAC) or untreated tumor cells. In marked contrast, when ST2^−/−^ spleen cells were assayed in the chip for their migratory response to treated melanoma cells, no significant displacement was observed towards either right (DAC/IL-33) or left (single treatment or no treatment) melanoma chambers (Fig. 6C-D). These results indicate not only that DAC and IL-33 synergized in promoting immune cell recruitment to the tumor site but also that IL-33/ST2 signaling is crucial for immune migratory response to DAC.

**Fig. 7:**
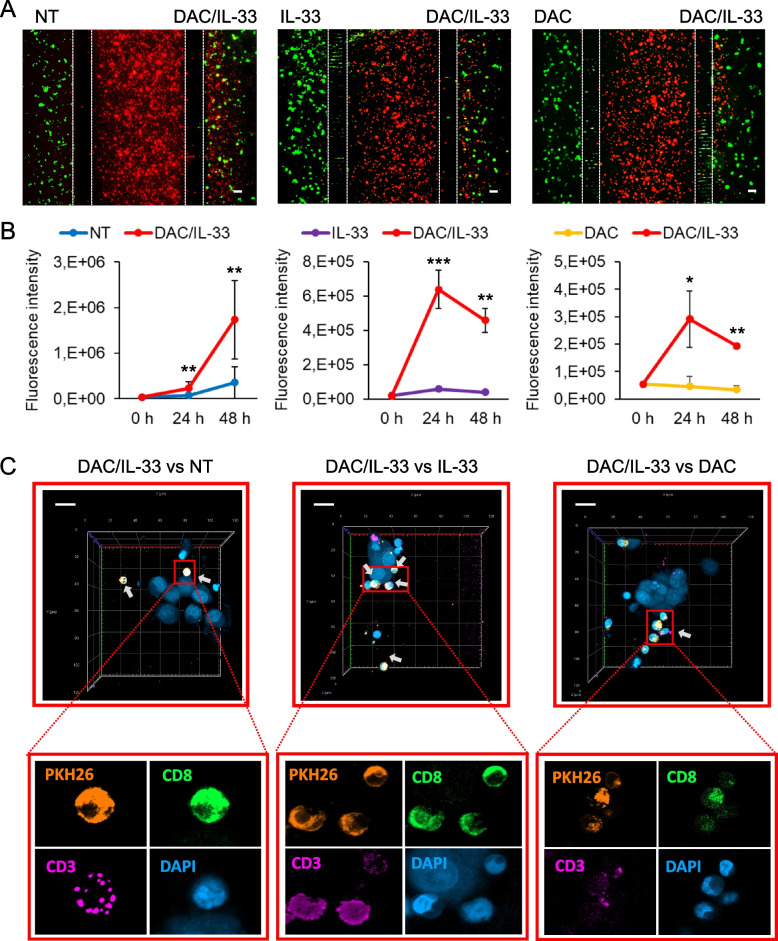
DAC/IL-33 increase the recruitment of PBMCs including CD8^+^ T cells towards A375M melanoma cells in a 3D microfluidic system. **A** Competitive migration assay of PBMCs towards DAC/IL-33 treated A375M melanoma cells in a 3D microfluidic chip. PKH26-labeled (red) PBMCs were loaded in the central chamber of microfluidic devices. PKH67-labeled (green) A375M melanoma cells embedded in Matrigel containing the treatments were placed in lateral chambers. In every device the condition DAC/IL-33 was confronted with not treated condition (NT, left), IL-33 alone (central) or DAC alone (right). Fluorescence images were obtained after 48 h of culture. Red fluorescence represents displacement of PBMCs towards melanoma chambers (green) containing the indicated treatments. Discontinued vertical white lines depict microchannel area. Scale bars = 100 mm. **B** Quantitative analysis of red fluorescence intensity in the tumor chambers reflecting the preferential migration PBMCs towards DAC/IL-33 treated vs untreated (left), IL-33 treated (middle) or DAC treated (right) melanoma cells. Data show the mean ± SD of multiple fields of at least 3 devices per experimental condition. *P< 0.05, ** *P*< 0.01, *** *P*< 0.001. **C** Confocal analysis showing a Z-stack view on XY plane mimicking the DAC/IL-33 treated melanoma chambers as opposed to untreated (left), IL-33 treated (middle) or DAC treated (right) after 48 of culture. This view details T lymphocytes (small sized DAPI^+^PKH26^+^CD3^+^ CD8^+^ cells) spatially interacting (white arrows) or not with A375M melanoma cells (DAPI^+^ cells, with large nuclei). Z-stack acquisition was performed by a 40X objective set up into the LSCM station. Bottom panel (red box) shows magnification microphotographs with the separate fluorescence channels used to distinguish T lymphocytes from the resting PBMCs and melanoma cells. Scale bars, 20 mm

Finally, to translate these observations in the human setting, we carried out the 3D microfluidic chip-based competitive assay with human A375M melanoma cells and PBMCs from healthy donors. A clear preferential migration towards the chamber containing DAC/IL-33 treated A375M, as opposed to single drug-treated (IL-33 or DAC) or untreated tumor cells was observed after 48 h of co-culture (Fig. 7A-B). Staining of PBMC migrated into the Matrigel chambers containing DAC/IL-33 treated A375M, showed the presence of several CD8 T cells in proximity of the tumor cells in all experimental conditions (i.e. vs NT, vs IL-33, vs DAC; Fig. 7C). In contrast, the few PBMC infiltrating the opposite chambers containing A375M cells either untreated (Supplementary Fig. 6), IL-33 treated (Supplementary Fig. 7) or DAC treated (Supplementary Fig. 8) showed no or rare CD8 T cells distant from tumor cells. Overall, these data demonstrate the cooperative action of DAC/IL-33 in promoting immune cell recruitment, including CD8 T cells.

## Discussion

Melanoma is one of the most immunogenic tumors, associated with high mutation burden and neo-antigen formation. For these features melanoma has high potential to elicit specific anti-cancer responses to immunotherapy targeting immune checkpoints, such as PD-1 and CTLA-4 [30–32]. However, due to the fact that not all patients respond to this therapy (i.e., 30–40% for anti-PD-1 monotherapy, increasing up to 60% for combined anti-PD-1 and anti-CTLA-4 immunotherapy) and considering the development of secondary resistance to immunotherapy, more efficient protocols of combined treatment are required. Previous studies from our group and others indicate that IL-33 exerts anti-tumor effects in melanoma models by tailoring the tumor immune microenvironment [2]. IL-33 is known to stimulate a wide set of immune cells such as CD8 T and NK cells [6, 33, 34], eosinophils [3, 4, 35–37], basophils [38], DC [5] and ILC2 [7, 39]. This suggests that this epithelial alarmin can be appointed as a valid immune modulator in combined therapeutic settings against melanoma.

In the present study, we demonstrate a co-operative action of DAC with exogenous and endogenous IL-33 signaling to promote tumor-immune crosstalk in melanoma. First, we show that a single DAC injection followed by 5 intratumoral injections of IL- 33 can remodel the TME ameliorating immunotherapy response to PD-1 blockade in melanoma-bearing mice. With respect to single treatments, DAC/IL-33 improved the survival of tumor-bearing mice although it did not significantly impact tumor growth. Of note, the most prominent effect of DAC/IL-33 occurred at the TME level. In this district, we observed a synergistic action in increasing the expression of Th1-related cytokines (i.e., granzyme B and IL- 12) and chemokines (i.e., CCL5). CCL5 is a potent chemoattractant for many cell types including monocytes, NK cells, T cells, eosinophils, mast cells and DC [40]. Accordingly, we observed a more prominent accumulation of CD4-T and CD8-T cells as well as eosinophils, but not G-MDSC, in the TME of mice exposed to the combo DAC/IL-33 treatment with respect to mice receiving single treatments or untreated. This immune scenario is consistent with the anti-tumorigenic activities of the combo treatment. In particular, IL-33 driven eosinophils are known to promote anti-tumor responses through the production of CD8 T cell-attracting chemokines [4, 41], release of extracellular vesicles [35] and direct cytotoxicity [3, 4, 37]. The superior ability of the combo DAC/IL-33 to elicit immune cell recruitment with respect to single treatments was further confirmed in organ-on-chip competitive assays. Through this approach, we could translate the findings of effector immune cell recruitment elicited by DAC/IL-33 also in the human system. In this setting, human A375M melanoma cells treated with DAC/IL-33 attracted more PBMCs, including many CD8 T cells, with respect to A375M cells exposed to single treatments or left untreated. Most importantly, DAC and IL-33 synergistically enhanced the expression of PD-1 within the TME and the percentages of PD-1 expressing tumor-infiltrating CD8 and CD4 T cells, resulting in improved in vivo response to PD-1 blockade. DAC/IL-33 and PD-1 mAb strongly synergized to increase tumor-infiltrating immune effector cells (i.e., CD4 T cells, CD8 T cells and eosinophils) and intratumoral expression of effector molecules (i.e., IFN-γ, granzyme B, perforin and TNF-α) which may account for therapeutic efficacy.

Epigenetic reprogramming can sensitize cancer cells to immune checkpoints blocking therapies through the upregulation of immunostimulatory cytokines (e.g. CXCL9 and CXCL10) that recruit T lymphocytes to the tumor site [17, 42]. Decitabine has been shown to increase tumor-infiltrating T cells and to upregulate the expression of immune checkpoints, such PD-1 and PD-L1, ameliorating the effectiveness of immune checkpoint blockade in colorectal cancer [43–45] and PDAC [46]. In melanoma, daily injections of guadecitabine, a dinucleotide prodrug of decitabine, in combination with anti-PD-1 and anti-CTLA-4 mAbs were found to reduce in vivo tumor growth [19]. In our melanoma model, the single treatment with DAC neither induced PD-1 in the TME nor increased the therapeutic response to anti-PD-1. This discrepancy may rely on the different treatment schedule employed in our experiments (i.e. single injection) with respect to other studies using repeated injections of the DNMTi. Expectedly, DAC exerted also direct anti-tumor effects, as demonstrated by inhibition of tumor aggregation of 3D spheroids and by cell viability reduction of mouse and human melanoma cells. Since DAC is considered an S-phase specific drug, because it is incorporated into the DNA during replication [47], its direct effect is likely attributable to an arrest of the cell cycle in G2/M-S phase and by the hypomethylation of tumor suppressor silenced genes. In contrast, IL-33 did not exert any direct action on melanoma cells and its combination with DAC did not further decrease tumor growth in either 2D or 3D settings, corroborating that the antitumor functions of this cytokine are attributable to the stimulation of immune response [2].

It was previously reported that the combination of IL-33 with PD-1 mAb prolongs the survival of leukemia-bearing mice [48] and PDAC mice [14] and stimulates anti-tumor responses in mouse models of Ret melanoma [13] and orthotopic lung cancer [49]. In apparent contrast with these findings, we found that IL-33 alone poorly induced PD-1 in the TME and did not ameliorated PD-1 blockade in melanoma-bearing mice. However, as opposed to prolonged systemic IL-33 injections employed by others, in our vivo studies we administered the cytokine intratumoral for 5 days before administering the PD-1 mAb, which may be insufficient to maintain persistent levels of PD-1 throughout the blockade therapy. Nevertheless, it is worth noting that intratumoral administration of IL-33 was as efficient as systemic delivery IL-33 [4] in inducing tumor-infiltrating eosinophils and CD8 T cells and in delaying tumor growth. This finding suggests that IL-33 acts locally in the TME to stimulate anti-tumor responses, as also observed by others [6, 7, 50, 51].

Another finding reported in this study is the requirement of IL-33/ST2 axis for immune recruitment to the TME and for in vivo anti-tumor efficacy of DAC. This was revealed by absence of therapeutic response to DAC treatment in ST2^−/−^ tumor-bearing mice and by 3D microfluidic chip experiments showing lack of migration of ST2^−/−^ immune cells towards DAC/IL-33 treated tumors. In vivo, the up-regulation of IL-33 and ST2 expression early after DAC administration (i.e., 3 days) was associated with up-regulation of T cell-attracting chemokines CCL5, CCL11, CXCL9 and the eosinophil-attracting chemokine CCL11, and accumulation of CD4-T, CD8-T cells and eosinophils in the TME of WT but not in ST2^−/−^ tumor-bearing mice. This finding is in agreement with previous reports showing that host derived IL-33 recruits and activates intratumoral CD8 T cells resulting in tumor growth control in PDAC [14] and colon cancer [52]. The effect of DAC, however, was transient since, at later time points (i.e., 7 days after DAC treatment), the levels of chemokines, T cells and eosinophils in the TME were comparable to those found in control mice (Fig. 2D-E). As a possible explanation for this transient phenomenon, we hypothesize that the biological activity of DAC-induced IL-33 may be eventually inhibited by oxidation [53] or proteolytic cleavage by apoptotic caspases [54] present in the TME, causing IL-33 inactivation at later time points. In this respect, the addition of recombinant IL-33 may sustain the recruitment of T cell and eosinophils for longer time.

In the TME, epithelial, endothelial, fibroblast-like and tumor cells can all be a source of IL-33 [55]. Although we cannot exclude that in vivo DAC induces the expression of the cytokine also in fibroblast and stromal cells, our in vitro experiments suggest that IL-33 is tumor-derived. This assumption is supported not only by up-regulation of IL-33 expression in mouse and human melanoma cells following exposure to DAC but also by the organ-on-chip experiments, which underscored a crosstalk between DAC exposed tumor cells and ST2-expressing immune cells, likely via tumor released IL-33. Several evidences indicate that the expression of IL-33 can be epigenetically regulated [56]. The histone deacetylase HDAC3 was shown to act as a transcriptional repressor of IL-33 in multiple sclerosis patients [57] and in a model of *Staphylococcus aureus*-aggravated skin inflammation associated to atopic dermatitis [58]. Moreover, in airway epithelial cells expression of IL-33 is regulated by histone H3 acetylation and trimethylation at specific sites of *IL33* gene promoter [59, 60]. Here, we show that DAC induces *IL33* promoter specific demethylation in both mouse and human melanoma cells. To our knowledge, this is the first evidence of a DNA methylation-mediated epigenetic regulation of IL-33 expression. Intriguingly, the observation that DAC positively modulates the expression of both IL-33 and ST2 in the TME of melanoma-bearing mice may suggest an epigenetic control of this axis.

## Conclusion

Overall, our studies underscore an important role for IL-33/ST2 in driving tumor-immune cross talk and indicate DAC as a positive regulator of this axis that can further increase anti-tumor effects of IL-33 in combinatorial approaches against melanoma, including the use of ICB. Decitabine and its derivatives have been FDA-approved for the treatment of hematological malignancies and are currently being investigated in clinical trials for solid cancers [61]. A better understanding of the molecular and immune mechanisms affected by DAC in the TME may provide rationale for novel combinatorial anti-cancer therapies. It has been shown that low-dose regimens of DAC can restore tumor immune control by reactivating multiple innate immune pathways, including type I IFN and immunogenic cell death pathways, especially in B16 melanoma [62]. In this respect, given the function of IL-33 as an alarmin released upon cell necrosis or damage it is not surprising that this cytokine is a target for DAC in the TME. Of note, such a remodeling of the TME via immune cell recruitment is central to overcome resistance to PD-1 checkpoint blockade. Moreover, due to the pleiotropic functions in the TME, IL-33 holds potential as an immune activator for combination therapies with DAC and possibly other DNMTi.

## Supplementary Information

Supplementary Material 1.

Supplementary Material 2

Supplementary Material 3: Supplementary Figure 1.* Bird’s eye view of the 3D microfluidic chip used for the competitive assay experiments.* (A) Schematic overview depicting the 3D structure of the microfluidic chip used. The overall chip structure with the loading wells and chambers is shown. Circular box illustrates a magnified image of cell chambers. (B) Illustration showing an exemplificative 3D loading of immune cells and tumor cells into the chip, depicting the estimated distribution of these cells in the various chip chambers. This image has been generated with the open-source CAD software Blender (version 4.3.1; https://www.blender.org/). (C-E) Representation of cell loading details and experimental condition for the competitive assay experiments, with specific references to the chamber’s loading definitions of the devices. Immune cells, loaded in the central chamber of the chip, are represented by either human PBMCs or mouse spleen cells. Tumor cells are loaded in side channels in presence of Matrigel, and are represented by either A375M for human organ-on-chip competitive assays or B16.F10 melanoma cells for mouse studies. Supplementary Figure 2. *Graphical representation of the mouse and human IL33 gene promoters assayed by MSP-qPCR.* Representation of a localization map for (A) mouse (Chromosome 19) and (B) human (Chromosome 9) *IL33* gene promoters analyzed by MSP-qPCR. Yellow rectangle depicts the amplicon of the examined promoter, generated by the forward and reverse primers (black arrows) used to study their methylation status. Chromosome maps delineated in the upper part of each figure are based on the conventional cytogenetic band nomenclature, and shows the position of gene promoters in mouse (A, *Il33* P1 promoter sequence in qC1 region inside the long arm of Chromosome 19) and human (B, *IL33* P promoter sequence in p24.1 region inside the short arm of Chromosome 9). Chromosome maps have been generated by the Genome Data Viewer (National Library of Medicine, https://www.ncbi.nlm.nih.gov/gdv/). Graphical maps of gene isoforms and their promoter sequences have been obtained by the ENSEMBL webpage tool (https://useast.ensembl.org/index.html) via the Gencode database version 46 (released in May 2024). P1, mouse *Il33* promoter sequence; P human *IL33* promoter sequence. Brown boxes, exons. Red boxes, promoter sequences. Supplementary Figure 3. *Effect of DAC and IL-33 on melanoma cells.* MTS cell viability assay. (A) B16.F10 melanoma cells were cultured alone (NT) or in the presence of rmIL-33 (100 ng/mL) and/or DAC at the indicated concentrations. Cell viability was evaluated at the indicated time points. Mean ± SD of three replicates is shown. (B) A375M melanoma cells were cultured alone (NT) or in the presence of rhIL-33 (100 ng/mL) and/or DAC at the indicated concentrations. Cell viability was evaluated at the indicated time points. Mean ± SD of five replicates is shown. *P< 0.05, **P< 0.01, ***P< 0.001, ****P< 0.0001. Supplementary Figure 4. * DAC and IL-33 alone or combined do not enhance the percentage of tumor-infiltrating T cells expressing LAG-3, Tim-3 or TIGIT.* C57BL/6 mice were injected subcutaneously with B16.F10 melanoma cells (0.8 x 10^5^) and then treated with DAC (1 mg/kg i.p.), IL-33 (0.4 mg/mouse i.t.) singly or combined. Control mice (CTR) received PBS. Ex vivo analyses were carried out on day 13. Percentage of tumor-infiltrating (A) CD8-T and (B) CD4-T cells expressing LAG-3, Tim-3 and TIGIT. Data represent the mean value of individual mice (*n*= 7) ± SD. Supplementary Figure 5. * PD-1 mAb does not synergize with DAC or IL-33 alone in reducing tumor growth in melanoma-bearing mice.* (A) Tumor growth in C57BL/6 mice implanted with B16.F10 melanoma cells (0.8 x 10^5^) treated with IL- 33 (0.4 mg/mouse i.t.), PD-1 mAb (250 m i.p.), singly or combined. (B) Tumor growth in melanoma-bearing mice after treatment with DAC (1 mg/kg i.p.) in combination or not with PD-1 mAb (250 m i.p.). Mean tumor area of individual mice (*n*= 7) ± SD is shown. Supplementary Figure 6. *3D overview of A375M NT chamber of the 3D microfluidic chip.* Confocal analysis acquired from untreated (NT) A375M-gel chamber 48 h after a competitive migration assay of PBMCs towards DAC/IL-33 treated vs NT A375M melanoma cells in a 3D microfluidic chip. The Z-stack view on XY plane shows absence of PBMCs (DAPI^+^PKH26^+^, small nuclei) in the NT A375M cell (DAPI^+^cells, large nuclei) chamber. Z-stack acquisition was performed by a 40X objective set up into the LSCM station. Scale bar, 20 mm. Supplementary Figure 7. *3D overview of A375M IL-33 chamber of the 3D microfluidic chip.* Confocal analysis acquired from IL-33-treated A375M-gel chamber 48 h after a competitive migration assay of PBMCs towards DAC/IL-33 treated vs IL-33-treated A375M melanoma cells in a 3D microfluidic chip. The Z-stack view on XY plane shows the presence of one single CD8 T cell (DAPI^+^PKH26^+^CD3^+^CD8^+^, small nuclei) spatially distant from A375M melanoma cells (DAPI^+^cells, large nuclei). Z-stack acquisition was performed by a 40X objective set up into the LSCM station. Bottom panel shows 5X magnification microphotographs of separate fluorescence channels. Scale bar, 20 mm. Supplementary Figure 8. *3D overview of A375M DAC chamber of the 3D microfluidic chip.* Confocal analysis acquired from DAC-treated A375M-gel chamber 48 h after a competitive migration assay of PBMCs towards DAC/IL-33 treated vs DAC-treated A375M melanoma cells in a 3D microfluidic chip. The Z-stack view on XY plane shows presence of rare PBMCs (DAPI^+^PKH26^+^cells, small nuclei) in the chamber with spatial interaction with A375M melanoma cells (DAPI^+^cells, large nuclei). Z-stack acquisition was performed by a 40X objective set up into the LSCM station. Bottom panel (red box) shows 5X magnification microphotographs of separate fluorescence channels showing no expression of CD3 or CD8 by infiltrated PBMCs. Scale bar, 20 mm.

## Data Availability

The data supporting the findings of this study are available from the corresponding author upon reasonable request.

## Abbreviations

*DAC*: Decitabine
DC: Dendritic cells
NK: Natural killer
G-MDSC: Granulocytic myeloid-derived suppressor cells
PBMCs: Peripheral blood mononuclear cell
ILC2: Type- 2 innate lymphoid cells
*TME*: Tumor microenvironment
*ICB*: Immune checkpoint blockade
*DNMTi*: DNA methyl transferase inhibitor
*GZMB*: Granzyme B
*PRF1*: Perforin
*WT*: Wild type
*CDDP*: Cisplatin
*DOXO*: Doxorubicin
*mRNA*: Messenger RNA
*qPCR*: Quantitative real time polymerase chain reaction
*MSP-qPCR*: Methylation-specific quantitative PCR
*PDAC*: Pancreatic ductal adenocarcinoma
