# Supplementary material for: Decitabine co-operates with the IL-33/ST2 axis modifying the tumor microenvironment and improving the response to PD-1 blockade in melanoma
Source: J Exp Clin Cancer Res. 2025 May 2;44:137. doi: 10.1186/s13046-025-03381-z (PMC12048997; doi:10.1186/s13046-025-03381-z)
Supplement: Supplementary file 1 — Supplementary Material 1. [file 13046_2025_3381_MOESM1_ESM.docx]

**Table S1. Primer pairs used for qRT-PCR.**

| **Human** |  |  |
| --- | --- | --- |
| **Gene** | **Accession number** | **Primer sequence (Forward/Reverse, 5' --> 3')** |
| *IL33* | NM_001314047 | AATCAGGTGACGGTGTTG |
|  |  | ACACTCCAGGATCAGTCTTG |
| *HPRT* | NM_000194.3 | TGACACTGGCAAAACAATGCA |
|  |  | GGTCCTTTTCACCAGCAAGCT |
| **Mouse** |  |  |
| **Gene** | **Accession number** | **Primer sequence (Forward/Reverse, 5' --> 3')** |
| *Il33* |  | Biorad #10025636 |
| *Lag3* | NM_008479.2 | TGTCTACAACTCACCGCGTC |
|  |  | CTCCAGACCCAGAACCTTGA |
| *Havcr2* | NM_134250.2 | TTGGAGTGGGAGTCTCCTGCT |
|  |  | ATCCTGACTGCTCCTGCATT |
| *Gzmb* | NM_013542.3 | GATCGGGAGTGTGAGTCCTAC |
|  |  | GAAAGCACGTGGAGGTGAAC |
| *Pd1* | NM_008798.3 | GATGCCCGCTTCCAGATCAT |
|  |  | AGGTCTCCAGGATTCTCTCTGTTA |
| *Tigit* | NM_001146325 | AAGCTCAGTGGCTCAGTTCC |
|  |  | TCAGGTTCCATTCCTGTGGC |
| *Cxcl10* | NM_021274.2 | CTCTCGCAAGGACGGTCCGC |
|  |  | TCCGGATTCAGACATCTCTGCTCAT |
| *Cxcl9* | NM_008599 | GGAGTTCGAGGAACCCTAGTGA |
|  |  | GTTCTTCAGTGTAGCAATGATTTCAG |
| *Ccl5* | NM_013653 | ATATGGCTCGGACACCACTC |
|  |  | GTGACAAACACGACTGCAAGA |
| *Prf1* | NM_011073.3 | GTGTCGCATGTACAGTTTTCG |
|  |  | TGTGGTAAGCATGCTCTGTG |
| *Tnf* | NM_013693.3 | CATCTTCTCAAAATTCGAGTGACAA |
|  |  | TGGGAGTAGACAAGGTACAACCC |
| *Ifna* | NM_008337 | TCAAGTGGCATAGATGTGGAAGAA |
|  |  | TGGCTCTGCAGGATTTTCATG |
| *Ccl11* | NM_011330.3 | CCCCAAGAAGAAGTGGGTCC |
|  |  | GGGCGACTGGTGCTGATATT |
| *St2* | NM_001025602 | GAATGGGACTTTGGGCTTTG |
|  |  | CAGGACGATTTACTGCCCTCC |
| *Il12b* | NM_001303244.1 | GGAAGCACGGCAGCAGAATA |
|  |  | AACTTGAGGGAGAAGTAGGAATGG |
| *Hprt* | NM_013556.2 | CTGGTGAAAAGGACCTCTCG |
|  |  | TGAAGTACTCATTATAGTCAAGGGCA |
