# Supplementary material for: Decitabine co-operates with the IL-33/ST2 axis modifying the tumor microenvironment and improving the response to PD-1 blockade in melanoma
Source: J Exp Clin Cancer Res. 2025 May 2;44:137. doi: 10.1186/s13046-025-03381-z (PMC12048997; doi:10.1186/s13046-025-03381-z)
Supplement: Supplementary file 2 — Supplementary Material 2 [file 13046_2025_3381_MOESM2_ESM.docx]

**Table S2. Primer pairs used for Methylation Specifi qRT-PCR**

| **Human** |  |  |
| --- | --- | --- |
| **Gene** |  | **Primer sequence (Forward/Reverse, 5' --> 3')** |
| *IL33 TFBS2 Meth* |  | TAGATTTATGAGAGTAGATTCGGAG |
|  |  | CTCTTTAAATAAAACCGATAACAAACG |
| *IL33 TFBS2 U-Meth* |  | AGATTTATGAGAGTAGATTTGGAG |
|  |  | CTTTAAATAAAACCAATAACAAACAAAA |
| **Mouse** |  |  |
| **Gene** |  | **Primer sequence (Forward/Reverse, 5' --> 3')** |
| *IL33 Meth* |  | GGTAGAGGAGTTGGGATATGAC |
|  |  | TTAACCTCCAAAACTAAAACTACCGT |
| *IL33 U-Meth* |  | GGTAGAGGAGTTGGGATATGAT |
|  |  | CTTAACCTCCAAAACTAAAACTACCAT |
