# Supplementary material for: Decitabine co-operates with the IL-33/ST2 axis modifying the tumor microenvironment and improving the response to PD-1 blockade in melanoma
Source: J Exp Clin Cancer Res. 2025 May 2;44:137. doi: 10.1186/s13046-025-03381-z (PMC12048997; doi:10.1186/s13046-025-03381-z)
Supplement: Supplementary file 3 — Supplementary Material 3: Supplementary Figure 1. Bird’s eye view of the 3D microfluidic chip used for the competitive assay experiments. (A) Schematic overview depicting the 3D structure of the microfluidic chip used. The overall chip structure with the loading wells and chambers is shown. Circular box illustrates a magnified image of cell chambers. (B) Illustration showing an exemplificative 3D loading of immune cells and tumor cells into the chip, depicting the estimated distribution of these cells in the various chip chambers. This image has been generated with the open-source CAD software Blender (version 4.3.1; https://www.blender.org/). (C-E) Representation of cell loading details and experimental condition for the competitive assay experiments, with specific references to the chamber’s loading definitions of the devices. Immune cells, loaded in the central chamber of the chip, are represented by either human PBMCs or mouse spleen cells. Tumor cells are loaded in side channels in presence of Matrigel, and are represented by either A375M for human organ-on-chip competitive assays or B16.F10 melanoma cells for mouse studies. Supplementary Figure 2. Graphical representation of the mouse and human IL33 gene promoters assayed by MSP-qPCR. Representation of a localization map for (A) mouse (Chromosome 19) and (B) human (Chromosome 9) IL33 gene promoters analyzed by MSP-qPCR. Yellow rectangle depicts the amplicon of the examined promoter, generated by the forward and reverse primers (black arrows) used to study their methylation status. Chromosome maps delineated in the upper part of each figure are based on the conventional cytogenetic band nomenclature, and shows the position of gene promoters in mouse (A, Il33 P1 promoter sequence in qC1 region inside the long arm of Chromosome 19) and human (B, IL33 P promoter sequence in p24.1 region inside the short arm of Chromosome 9). Chromosome maps have been generated by the Genome Data Viewer (National Library of Medicin [file 13046_2025_3381_MOESM3_ESM.pptx]

## Slide 1
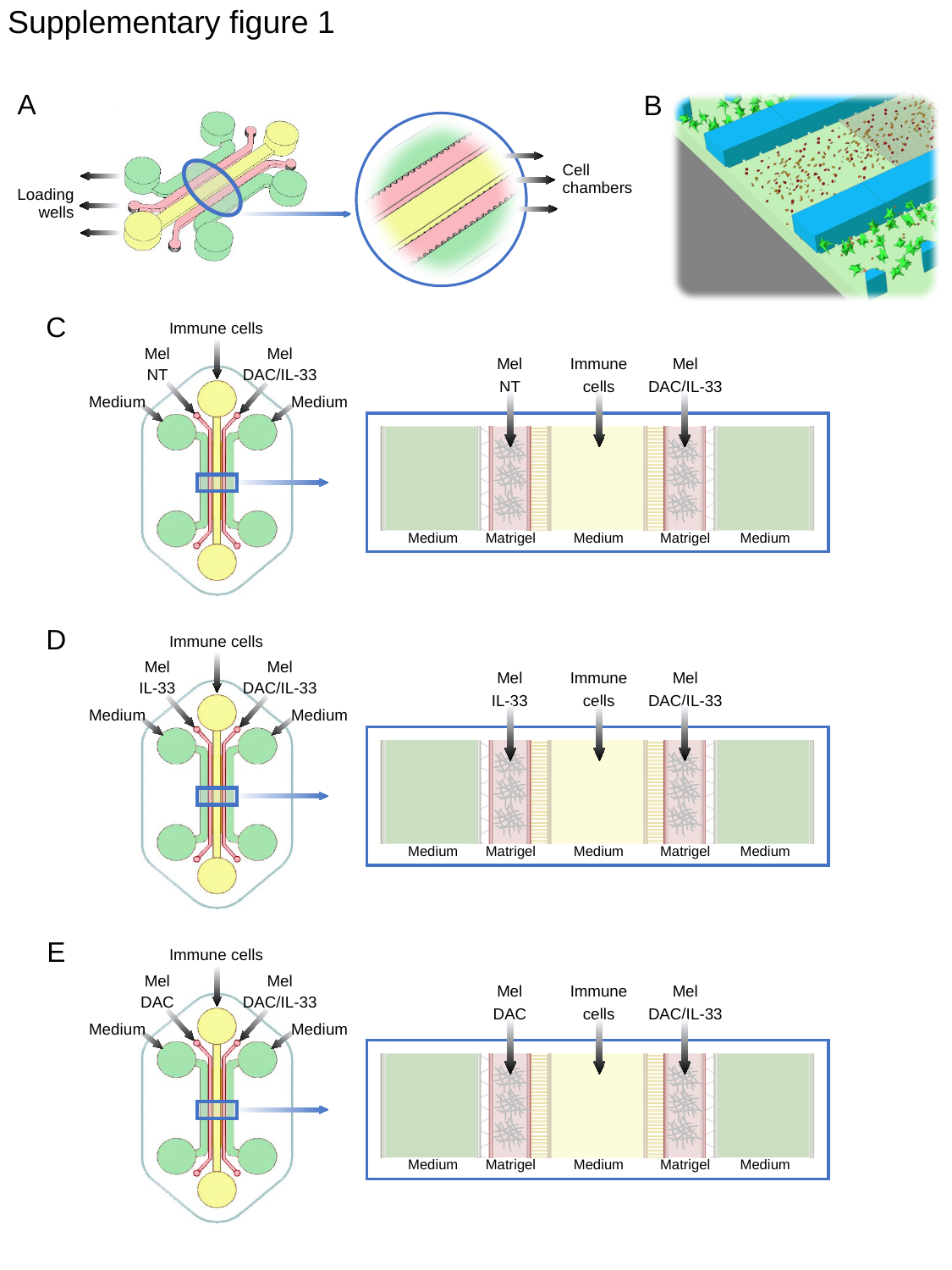

Supplementary figure 1
A
Cell
chambers
Loading
wells
B
C
Immune cells
Mel
NT
Mel
DAC/IL-33
Mel
NT
Immune
cells
Mel
DAC/IL-33
Medium
Medium
Medium
Matrigel
Medium
Matrigel
Medium
D
Immune cells
Mel
IL-33
Mel
DAC/IL-33
Mel
IL-33
Immune
cells
Mel
DAC/IL-33
Medium
Medium
Medium
Matrigel
Medium
Matrigel
Medium
E
Immune cells
Mel
DAC
Mel
DAC/IL-33
Mel
DAC
Immune
cells
Mel
DAC/IL-33
Medium
Medium
Medium
Matrigel
Medium
Matrigel
Medium

## Slide 2
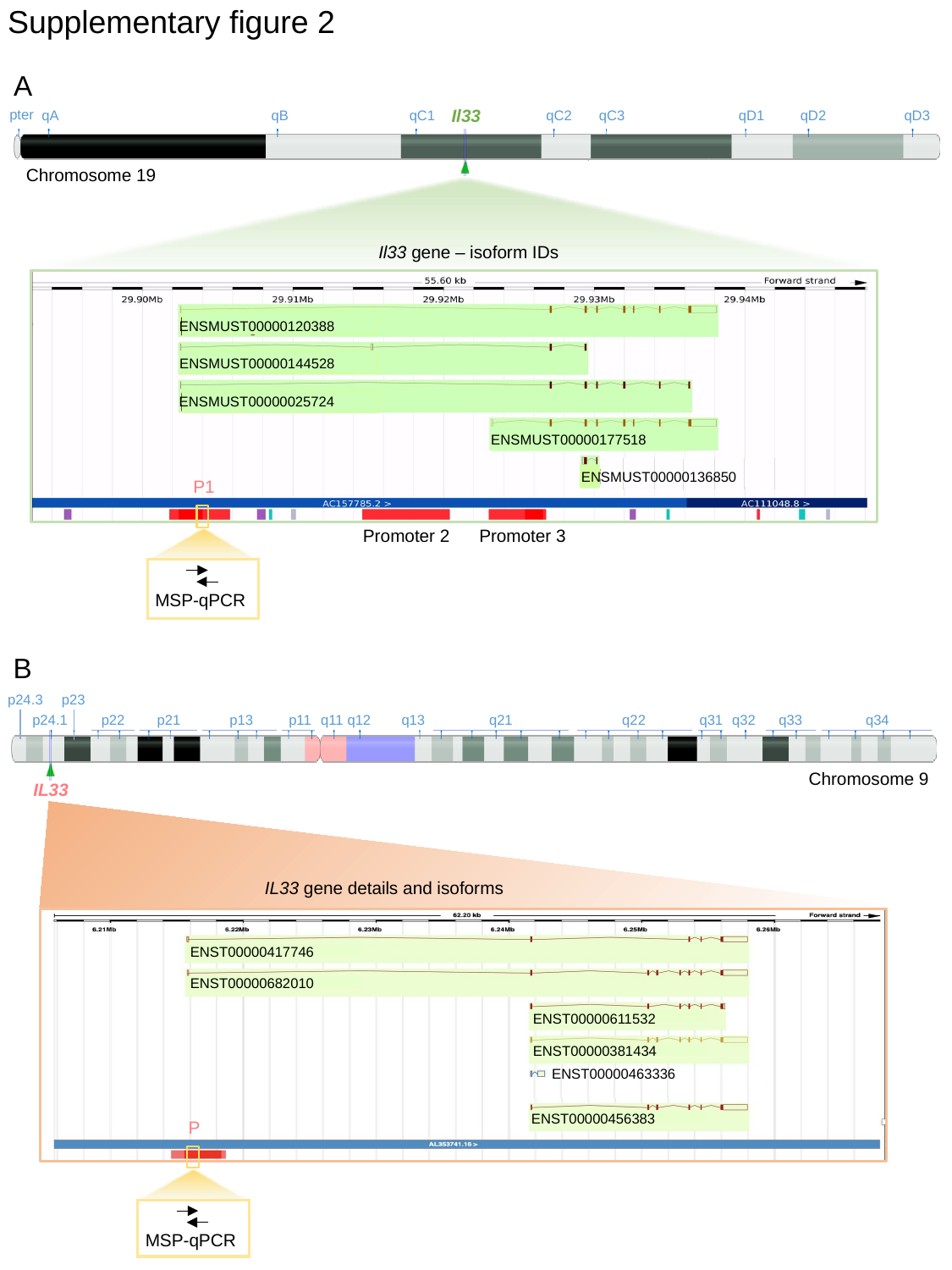

Supplementary figure 2
A
pter
qA
qB
qC1
qC2
qC3
qD1
qD2
qD3
Il33
Chromosome 19
Il33 gene – isoform IDs
D
ENSMUST00000120388
ENSMUST00000144528
ENSMUST00000025724
ENSMUST00000177518
ENSMUST00000136850
P1
Promoter 2
Promoter 3
MSP-qPCR
B
p24.3
p23
q34
q33
q22
q21
q32
q13
q12
q11
p11
p13
p22
p21
p24.1
q31
Chromosome 9
IL33
IL33 gene details and isoforms
ENST00000417746
ENST00000682010
ENST00000611532
ENST00000381434
ENST00000456383
ENST00000463336
P
MSP-qPCR

## Slide 3
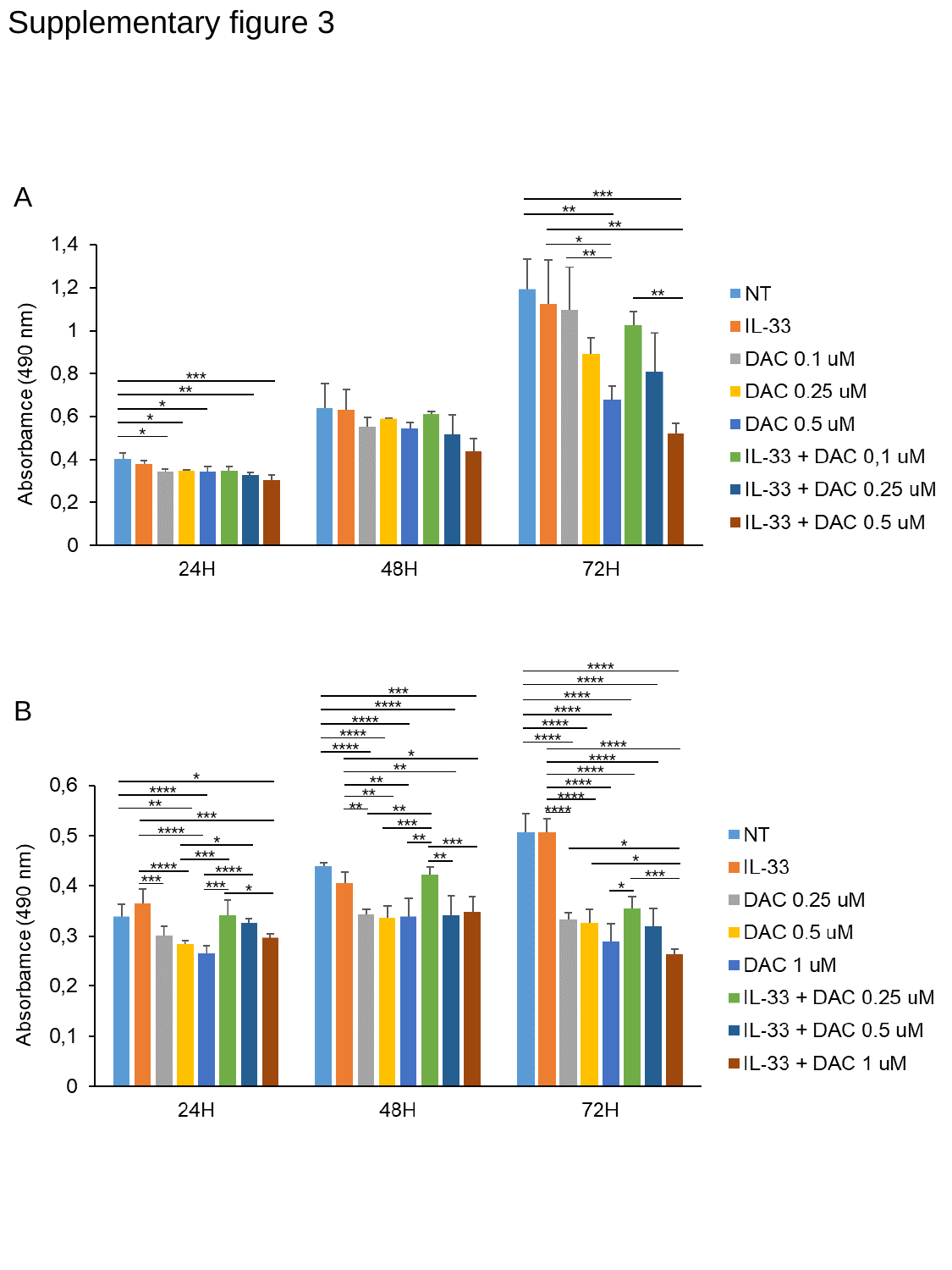

Supplementary figure 3
A
***
**
**
*
**
**
***
**
*
*
*
****
****
***
****
****
****
****
****
****
****
****
****
*
****
**
****
*
**
****
****
**
****
**
**
**
****
***
***
****
**
*
***
*
***
**
*
****
****
***
***
***
*
*
B

## Slide 4
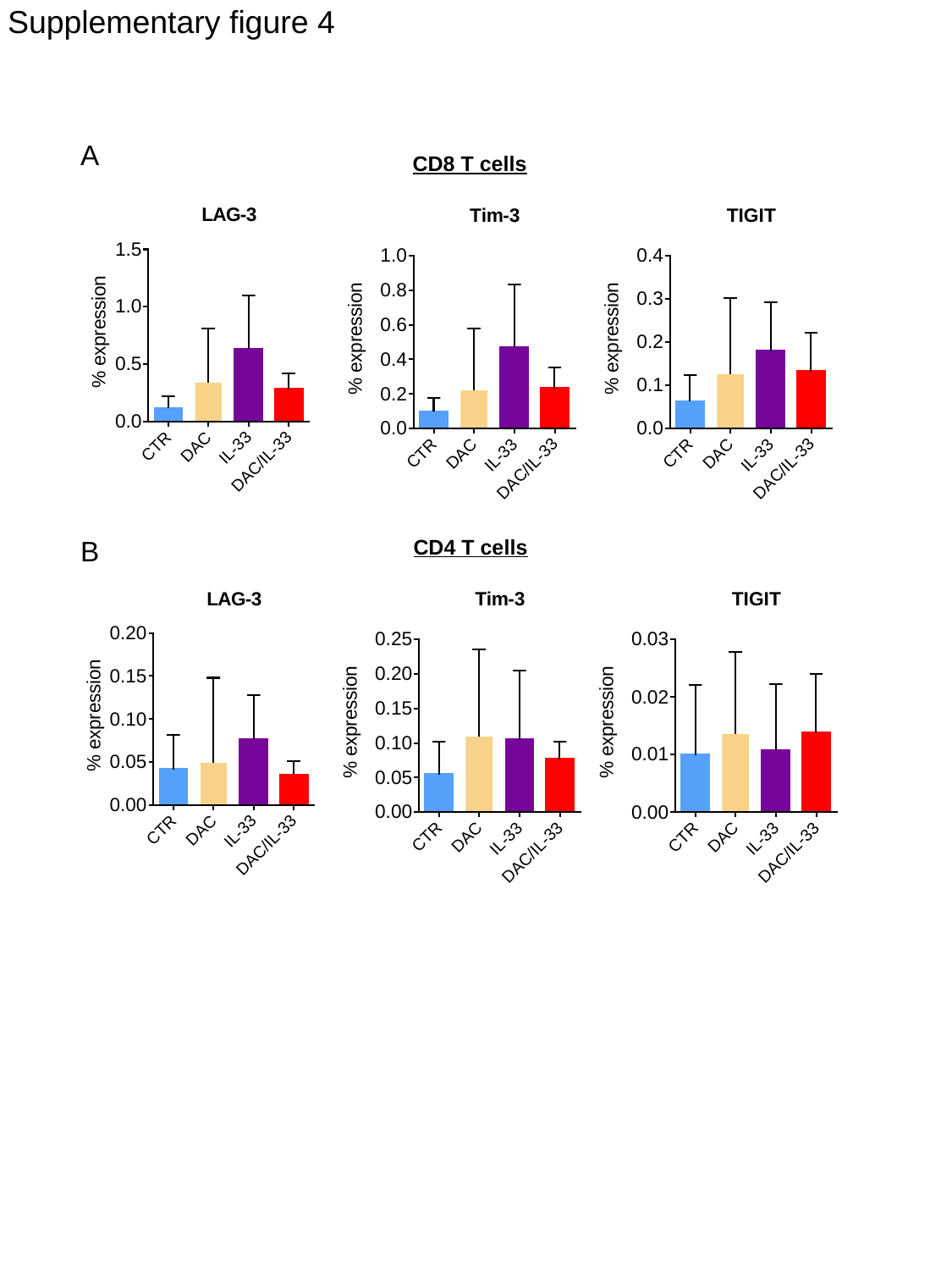

Supplementary figure 4
A
CD8 T cells
CD4 T cells
B

## Slide 5
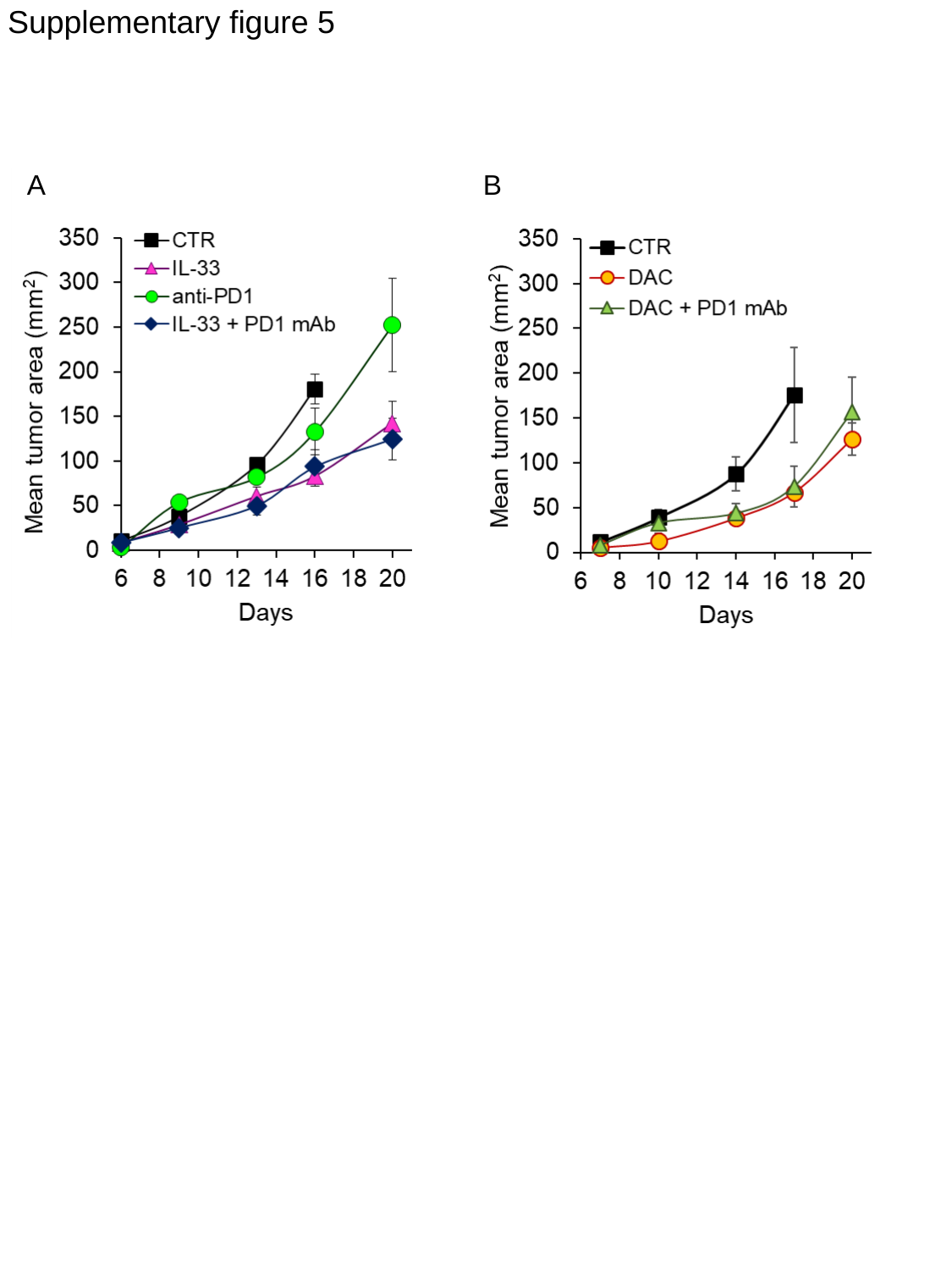

Supplementary figure 5
A
B

## Slide 6
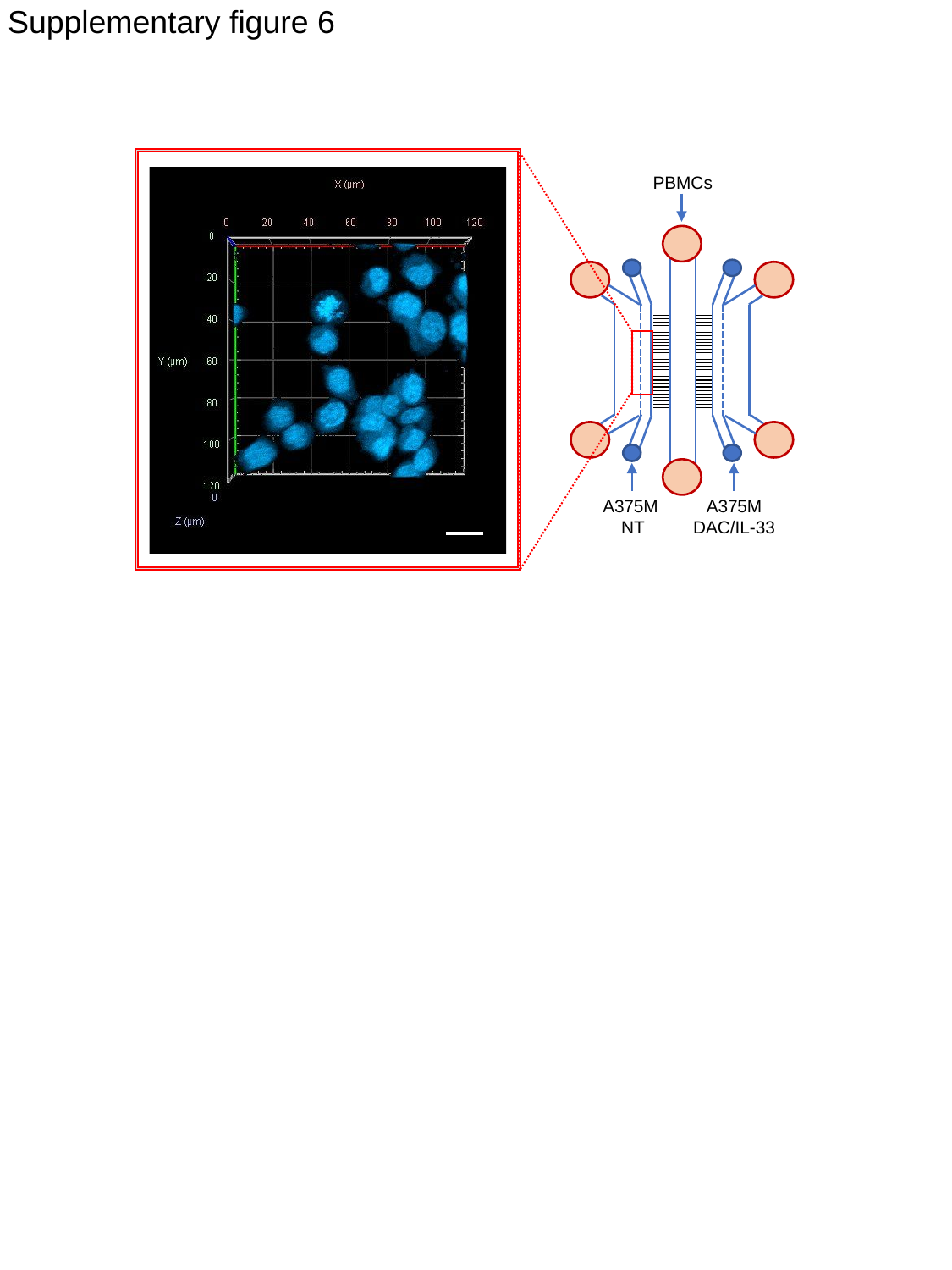

Supplementary figure 6
PBMCs
A375M
NT
A375M
DAC/IL-33

## Slide 7
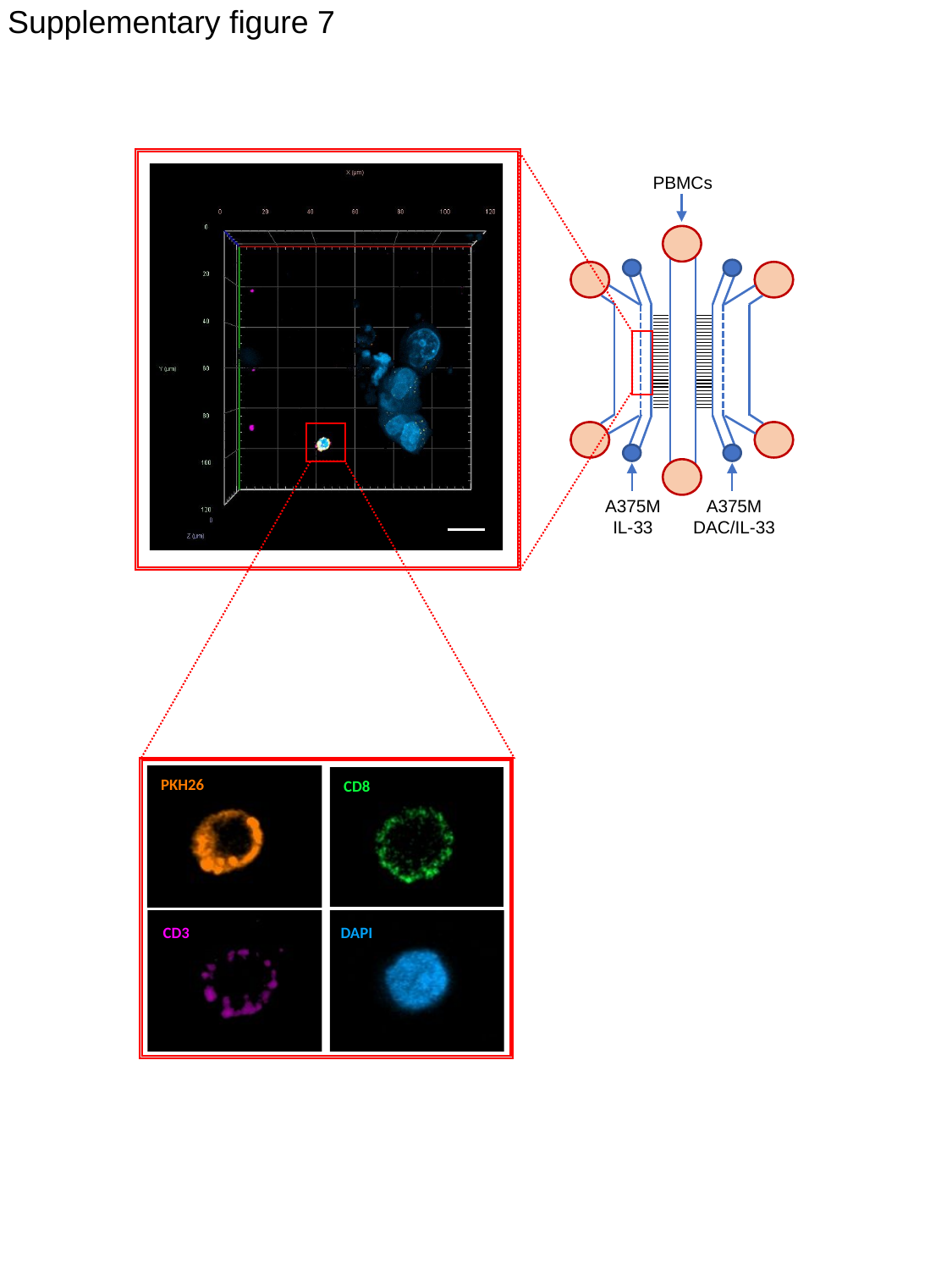

Supplementary figure 7
PBMCs
A375M
IL-33
A375M
DAC/IL-33
PKH26
CD8
DAPI
CD3

## Slide 8
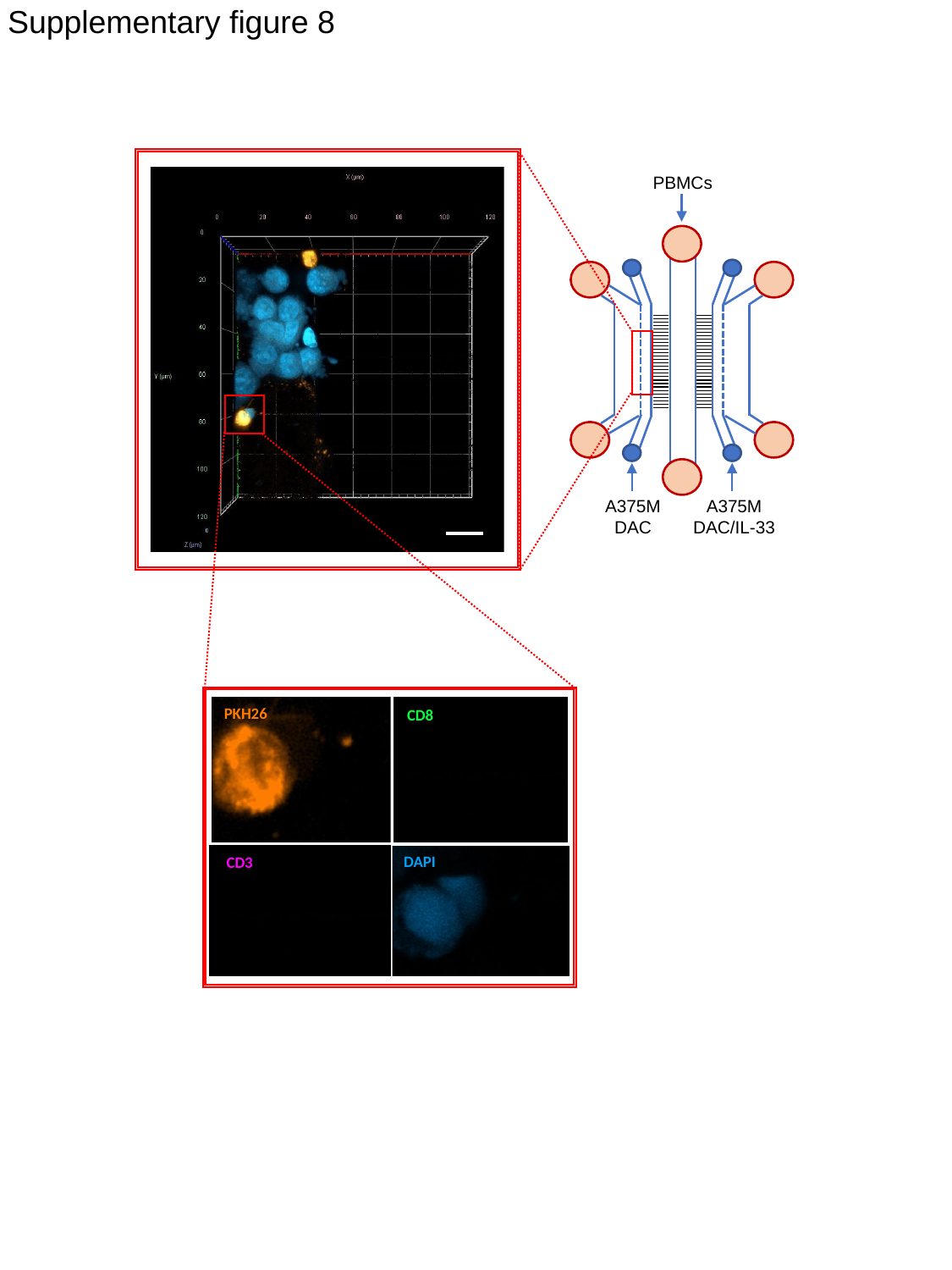

Supplementary figure 8
PBMCs
A375M
DAC
A375M
DAC/IL-33
PKH26
CD8
DAPI
CD3
